# Overt and implicit prosody contribute to neurophysiological responses previously attributed to grammatical processing

**DOI:** 10.1038/s41598-022-18162-3

**Published:** 2022-08-30

**Authors:** Anastasia Glushko, David Poeppel, Karsten Steinhauer

**Affiliations:** 1 Centre for Research on Brain, Language and Music, Montreal, Canada; 2 Department of Psychology, New York University, New York City, NY USA; 3 Ernst Struengmann Institute for Neuroscience, Frankfurt, Germany; 4 Center for Language, Music, and Emotion (CLaME), New York, USA; 5 School of Communication Sciences and Disorders, McGill University, 2001 McGill College Avenue, Unit 800, Montreal, QC H3A 1G1 Canada

**Keywords:** Cognitive neuroscience, Language

## Abstract

Recent neurophysiological research suggests that slow cortical activity tracks hierarchical syntactic structure during online sentence processing. Here we tested an alternative hypothesis: electrophysiological activity peaks at constituent phrase as well as sentence frequencies reflect cortical tracking of overt or covert (implicit) prosodic grouping. Participants listened to series of sentences presented in three conditions while electroencephalography (EEG) was recorded. First, prosodic cues in the sentence materials were neutralized. We found an EEG spectral power peak elicited at a frequency that only ‘tagged’ covert, implicit prosodic change, but not any major syntactic constituents. In the second condition, participants listened to a series of sentences with overt prosodic grouping cues that either aligned or misaligned with the syntactic phrasing in the sentences (initial overt prosody trials). Following each overt prosody trial, participants were presented with a second series of sentences lacking overt prosodic cues (instructed prosody trial) and were instructed to imagine the prosodic contour present in the previous, overt prosody trial. The EEG responses reflected an interactive relationship between syntactic processing and prosodic tracking at the frequencies of syntactic constituents (sentences and phrases): alignment of syntax and prosody boosted EEG responses, whereas their misalignment had an opposite effect. This was true for both overt and imagined prosody conditions. We conclude that processing of both overt and covert prosody is reflected in the frequency-tagged neural responses at sentence constituent frequencies. These findings need to be incorporated in any account that aims to identify neural markers reflecting syntactic processing.

## Introduction

Language comprehension involves a variety of cognitive mechanisms for processing multiple types of information, from auditory perception to integration of words’ semantic content with the grammatical structure of sentences. While some of these processing mechanisms have parallels across the animal kingdom^1^, building and processing syntactic structures has been suggested as a unique element of human language that distinguishes it from communication in other animals^2,3^. According to syntactic theories^4,5^, phrase structure is built from smaller linguistic elements that are combined into increasingly larger units (i.e., from words/morphemes to phrases to sentences), creating a hierarchical structure of grammatical constituents. However, whether this theoretical framework can help describe how the human brain processes language in real time remains controversial^6^. Psycholinguistic and neurolinguistic studies attempting to demonstrate the cognitive processing of hierarchically represented phrasal structures have typically used rather unnatural tasks (such as ‘click’ detection^7,8^) or inferred neurocognitive parsing mechanisms from processing of syntactic errors^9,10^. This work often produced ambiguous data that could alternatively be explained in terms of semantic or prosodic processing that takes place in parallel to, but is distinct from, syntactic processing^11^.

Several recent studies provided preliminary fMRI and electrophysiological data on brain responses to syntactic phrase boundaries in grammatical sentences^12–15^. The challenges of distinguishing syntactic processing effects per se from those that only appear to be syntactic explain why the recent magnetoencephalographic (MEG) findings by Ding et al.^14^ have been widely perceived as an important new approach to test cortical tracking of hierarchical sentence structures in human listeners^16,17^. Ding and colleagues’ experiments employed grammatical sentences, used a relatively natural task (listening to connected speech; detecting implausible sentences), and—importantly—explicitly addressed some alternative accounts. One such account was a prosodic one: the authors ruled out the contribution of overt prosody—i.e., of suprasegmental phonological features including sentence melody and stress patterns^18^—from contributing to their results. Their spoken sentence materials were stripped of tonal pitch and sound intensity changes, and all words within sentences had equal length, ensuring no acoustic cues could mark syntactic boundaries. Despite these precautions, however, it is still possible that Ding and colleagues’ findings might be strongly influenced by covert (or implicit) prosody. Covert prosody refers to subvocally activated prosodic representations (rhythm, intonation, focus etc.) during silent reading^19^, sometimes named the “inner voice”. It has also been demonstrated in other settings, such as in speech perception^20^ when overt prosodic cues were artificially removed from the stimuli. The present study was designed to test this hypothesis. Before outlining our specific approach, we briefly summarize Ding and colleagues’ findings and motivate why covert prosody may have played a role in eliciting such neural response patterns.

To demonstrate hierarchical constituent-driven processing, Ding and coauthors^14^ investigated periodic cortical activity using the ‘frequency tagging’ technique. This method can be used to ‘tag’ language characteristics requiring either (a) low-level stimulus-driven (‘bottom-up’) or (b) higher-level cognitively driven (‘top-down’) processing mechanisms. The authors presented participants with concatenated sequences of spoken four-syllable sentences (with no breaks between sentences), in which each word consisted of one syllable and lasted exactly 250 ms (making each sentence one second long). They found a stimulus-driven 4 Hz rhythm in listeners’ MEG signals. A 4 Hz peak in the MEG frequency spectrum was found even when English speakers listened to sentences in Mandarin Chinese, demonstrating that this ‘bottom-up’ cortical rhythm was elicited by the 4 Hz syllable rate of the acoustic signal (envelope tracking), independent of language comprehension (in line with Howard & Poeppel^21^). However, when Chinese and English participants listened to 4-syllable sentences in their native language, the MEG spectrum was characterized by two additional peaks—at 1 Hz (corresponding to the sentence rate) and 2 Hz (corresponding to the phrase rate). These two lower frequency effects did not correspond to any acoustic rhythms in the speech signal (nor, based on several control experiments, follow from statistical contingencies between words) and must, therefore, reflect cognitively driven ‘top-down’ brain activity related to understanding and structuring the utterances. In fact, these data were taken to demonstrate the human brain’s ability to track syntactic constituents concurrently at multiple distinct levels of the linguistic hierarchy. In both English and Mandarin Chinese, the sentences had been designed such that the first two syllables always created a syntactic noun phrase (NP; e.g., “new plans”), while the last two words created a syntactic verb phrase (VP; e.g., “give hope”). NP and VP in this “2 + 2” structure were separated by the sentence’s largest syntactic boundary in mid-sentence position (e.g., “New plans | give hope”). The authors interpreted the 1 Hz power peak to reflect parsing of the entire sentence (i.e., the largest syntactic constituent), and the phrase-level (2 Hz) peak to represent cortical tracking of the two syntactic units at the next level of the syntactic hierarchy (i.e., NP and VP). This interpretation was supported by an additional condition (in Chinese only) showing that the 2 Hz peak (but not the 1 Hz peak) disappeared when the largest syntactic boundary was placed after the first one-syllable word (“1 + 3” structure), thus separating two syntactic constituents of unequal length (1 syllable + 3 syllables, as in “fry | to-ma-toes”). Since all words used in these experiments were recorded separately, had identical length, and their pitch and sound intensity were held constant, Ding et al.^14^ provided strong evidence for cortical top-down mechanisms in online speech processing. A computational model links these findings to the larger question of composition in syntactic structures, and the construction of arguments more broadly^22^, lending support to the structure building interpretation.

However, there are alternative interpretations. Covert (or implicit) prosody processing is another top-down mechanism that has been shown to play an important role in real-time sentence processing^23,24^. The role of implicit prosody in the elicitation of neurophysiological power peaks at frequencies of syntactic units remains largely unknown. In other words, prosodic processing is not limited to bottom-up mechanisms driven by overt acoustic cues in the speech signal. Instead, readers have been found to systematically activate covert (implicit) prosodic patterns during silent reading, such as prosodic boundaries that group words into prosodic phrases^19,23,25^. For example, electroencephalographic (EEG) studies in English, German, Korean, and Mandarin Chinese have shown that both listeners and readers across languages reliably elicit a specific brain response for prosodic phrasing (i.e., the Closure Positive Shift, or CPS), irrespective of whether the phrasing pattern was induced by overt prosodic cues in the speech signal or covertly, using visual signs and other triggers during silent reading^26^. The CPS brain response in readers was triggered by punctuation marks like commas^26–28^, by long syntactic phrases that induce prosodic boundaries^29^, and by an instruction asking participants to imagine prosodic boundaries at specific positions while silently reading sentences^24^. Similar CPS findings reflecting prosodic top-down mechanisms have then been reported for speech processing as well, especially in the absence of overt prosodic cues^20^, i.e., for the kind of sentence materials used in Ding et al.’s^14,30^ frequency tagging studies. This top-down prosodic chunking often mirrors the reader’s or listener’s initial syntactic analysis, so mentally imposed prosodic phrases may directly correspond to syntactic phrases^20,29,31^. In this case, one could argue that brain responses related to prosodic phrasing are still driven by—and ultimately dependent on—syntactic processes. However, syntax and prosody do not always have a one-to-one mapping, as non-syntactic factors, including word or phrase length and the symmetry (or balance) of prosodic sister phrases, also play an important role in generating prosodic boundaries^23,25,32,33^. Phrase length, semantic coherence, and information structure cues can lead to the placement of prosodic breaks at positions where major syntactic breaks are absent^34–37^. Moreover, it is fair to assume that covert prosodic phrasing patterns mirror the high variability of prosodic realizations seen in speech production, with many prosodic boundaries being optional^38,39^ and inserted, for instance, driven by individual working memory capabilities^40^.

Thus, a given syntactic structure is often compatible with multiple distinct prosodic groupings, and a given prosodic structure may be applicable to multiple syntactic structures. For example, the sentence “[John]_**NP**_ [likes [big trees]_NP_]_**VP**_” has a 1 + 3 syntactic structure, where the monosyllabic subject NP John is followed by a 3-word VP (consisting of the verb likes and the object NP big trees). Prosodically, however, a 2 + 2 grouping (John likes | big trees) would be perfectly acceptable, and would meet the prosodic ‘symmetry’ constraint by creating two prosodic phrases of identical length^31^. Applied to Ding and colleagues’ materials, these considerations point to a confound between syntax and covert prosody in their study. Their 2 + 2 syntactic structure (e.g., “New plans | give hope”) is highly compatible with a 2 + 2 prosodic structure, placing both the syntactic and the prosodic boundary in mid-sentence position. In contrast, their 1 + 3 syntactic structure consisting of a monosyllabic verb and a trisyllabic object NP (“fry to-ma-to”, or “drink Oo-long tea”) is incompatible with a mid-sentence prosodic boundary, because it would separate syllables belonging to the same word (“fry to | ma-to”; “drink Oo | long tea”). Importantly, this is a lexical rather than syntactic reason. As a consequence, in both sentence structures, virtually the only possible prosodic grouping into two phrases happens to be identical to the syntactic phrasing. In other words, it is possible that the electrophysiological peaks observed by Ding et al.^14,30^ at syntactic phrase boundaries were instead elicited by coinciding prosodic phrase boundaries, which however cannot be argued to simply reflect syntactic structure. In addition, the entire 4-word utterance in all cases would correspond to the largest prosodic group (a so-called ‘intonational phrase’), which would thus provide a prosodic account for the sentence-level 1 Hz peak as well. As some lexical knowledge is necessary to identify both syntactic boundaries and possible prosodic boundary positions (e.g., between words, but not between syllables of the same word), both a prosodic and a syntactic processing account could equally explain Ding and colleagues’ neurophysiological power peaks at boundary positions in proficient users of a given language—as well as the absence of these peaks in listeners unfamiliar with the language.

The notion of covert prosodic phrasing becomes especially relevant when it comes to frequency tagging studies, where sentences are typically presented in a blocked design, such that a given trial of, say, 12 sentences contains either only 2 + 2 sentences or only 1 + 3 sentences. This way, listeners could quickly develop a covert prosodic template during the first few sentences and then apply this template to the remaining sentences of a trial, thereby eliciting the 2 Hz peak in 2 + 2 but not in 1 + 3 sentences. The initial motivation for generating these prosodic groupings can either be syntactic or non-syntactic in nature, but—as shown above—lexical rather than syntactic reasons seem to prevent a 2 + 2 prosodic grouping in 1 + 3 syntactic structures used by Ding and colleagues.

Crucially, even if the ultimate reason for the elicitation of frequency peaks in Ding et al.’s^14^ work and the range of subsequent studies were attributable to hierarchical syntactic processing as argued, it is important to know if (i) the syntax-related brain processes themselves are being tracked with this measure, or whether (ii) the cortical tracking of hierarchical linguistic structures is generally mediated by—and thus dependent on—the activation of implicit prosody. A third possibility is that (iii) both syntactic and prosodic phrasing are reflected by distinct peaks during frequency tagging. To distinguish among these three alternative accounts, first, one would have to disentangle syntactic and prosodic structures in the sentence materials. Second, one could actively manipulate the presence versus absence of prosodic information as well as syntactic or prosodic task requirements. Third, in the data analysis, one should also look at potential differences in the scalp distribution of frequency peaks. There exists at least some preliminary evidence that syntactic and prosodic processing are subserved by distinct neural circuits in the brain that may differentially affect the topography of EEG effects. On the one hand, syntax seems to be more strongly associated with brain structures in the left hemisphere, whereas prosody has been linked to the right hemisphere^41–43^. On the other hand, while syntactic processing is often viewed to involve Broca’s area (in the left inferior frontal cortex) as well as the dorsal stream and structures in the temporal lobe^42^, some fMRI work has identified right anterior circuits for prosody perception and left anterior circuits for prosody production^44^. These latter findings are potentially relevant, as overt prosodic processing of speech prosody is primarily based on perception, whereas covert (implicit) prosodic phrasing shares aspects of (subvocal) prosody production^45^. Importantly, since both left and right hemisphere circuits for prosody were found in anterior structures^44^, their contribution to frequency peaks may have a more frontal distribution than that of syntactic phrasing. As we will see, the present study attempts to use all three approaches to disambiguate prosodic from syntactic phrasing.

To summarize, given the potential confounds between syntactic and covert prosodic phrasing in Ding and coauthors’ materials, it is possible that their sentence (1 Hz) and phrasal (2 Hz) MEG power peaks do not exclusively reflect the hierarchical levels of syntactic structure, but rather—at least to some extent—the covert prosodic grouping of words.

## Present study

**Figure 1:**
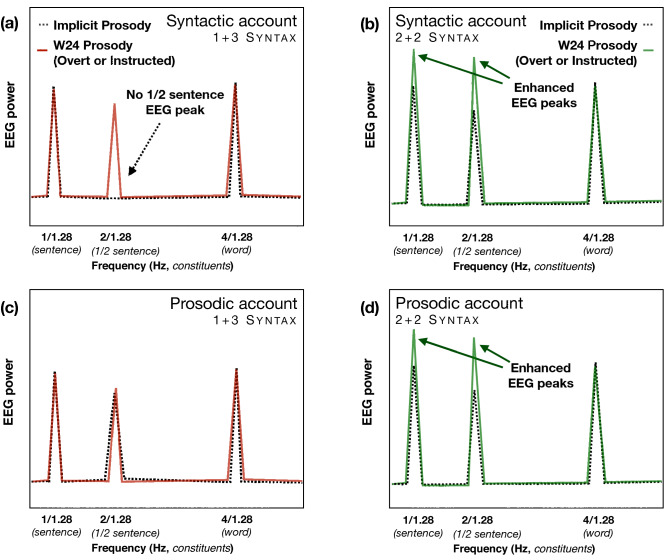
Predictions for the Implicit, Overt, and Instructed Prosody experimental conditions based on different theoretical assumptions. The top row represents the syntactic account of Ding et al.^14^ findings (**a**,**b**), while the predictions driven by the prosodic account are in the bottom row (**c**,**d**). If covert prosody does not play a role in the elicitation of EEG power peaks at syntactic constituent frequencies when overt prosodic cues are neutralized (i.e., Implicit Prosody condition), no ½ sentence peak (at the frequency of 2^[words]^/1.28^[0.32 s × 4 words]^) is expected for the 1 + 3 Syntax condition [dotted line in (**a**)]. In contrast, in the 2 + 2 Syntax condition, this peak would be present [dotted line, (**b**)]. Alternatively, if the ½ sentence peak can be accounted for by prosody, both syntactic structures would elicit it in the Implicit Prosody conditions (**c**,**d**). In the Overt and Instructed Prosody conditions (i.e., W24 Prosody in which sound intensity and pitch on Words 2 and 4 in a sentence are increased), we predicted, independently of the account, to see an interaction between syntactic and prosodic structures: when syntax and prosody are more strongly aligned, we expected to see enhanced EEG responses at sentence constituent frequencies (**b**,**d**). When they are less aligned, this effect would be significantly weaker or non-existent (**a**,**c**). Note that we only depicted the expected responses at sentence constituent frequencies, which will, in real data, be always accompanied by some level of noise and, potentially, harmonic neural activity.

To test the hypothesis that covert prosody contributes to the 1 Hz (sentence-level) and 2 Hz (phrasal frequency) peaks attributed to syntactic processing, we present an EEG experiment with German sentence materials that unconfounds syntactic and prosodic phrasing. Similar to Ding et al.^14^, we created 2 + 2 and 1 + 3 syntactic structures. However, in contrast to their materials, our 1 + 3 Syntax condition was still compatible with a 2 + 2 prosodic grouping (similar to the sentence example provided above, i.e., John likes big trees). In our first Implicit Prosody condition, we adopted Ding and coauthors’^14^ paradigm and presented series of 4-word sentences without any prosodic information. We predicted that if syntax alone was responsible for the sentence- and phrase-level EEG peaks, the 1 + 3 Syntax condition should replicate their original findings and not elicit the phrasal frequency peak (see Fig. 1a). However, if covert prosody was involved, the 1 + 3 Syntax condition could now elicit both the sentence and the phrasal (½ sentence) peaks (Fig. 1c). Further, in our Overt Prosody and Instructed Prosody conditions, we created prosodic contours that were expected to differentially interact with the two syntactic structures (2 + 2 and 1 + 3, respectively; for an illustration, see Fig. 1). These prosodic contours were applied to the sentences from our first (Implicit Prosody) condition, either overtly by modulating the auditory sentence materials, or covertly by instructing participants to imagine a specific prosodic contour while listening to sentences without overt prosodic cues. Our expectation was that both overt prosody and instructed (covert) prosody manipulations should increase at the very least the phrasal frequency peaks in sentences with a 2 + 2 syntactic structure, but not in those with a 1 + 3 structure. Such a differential pattern would reflect an interaction between syntactic and prosodic processing often reported in previous behavioural and EEG research^46,47^.

## Methods

### Participants

Twenty-six participants (age range: 19–45 years, mean age = 27, age SD = 6; 15 females, 11 males) took part in the study. This number is somewhat larger than in the original studies by Ding and colleagues, with eight participants in each of their MEG experiments^14^ and 16 participants in their EEG study^30^; it is also comparable to the recent MEG study testing the lexico-semantic account^48^ of Ding et al.’s^14^ findings where the number of participants was 27. All participants had acquired German language from birth and considered it their dominant language. They were recruited and tested at McGill University in Montreal, most of them visiting Canada for work-and-travel purposes. The inclusion criteria for the study were the absence of neurologic or psychiatric disorders and hearing impairments, as well as normal or corrected vision. Participants provided written informed consent and received monetary compensation ($20/h) for their time.

We assessed handedness using the Edinburgh Handedness Inventory ensuring all participants were right-handed^49^. Participants filled out detailed in-house questionnaires about their language background and musical expertise ensuring all of them were native speakers of German and non-musicians. All parts of the study were approved by McGill’s Faculty of Medicine Institutional Review Board (IRB) prior to data collection; all methods were performed in accordance with the IRB’s guidelines and regulations.

### Materials

Speech synthesis. The four-word German sentences used in the experiment were synthesized word-by-word with a built-in Apple synthesizer (the Anna voice). All words were monosyllabic, and their speech signals were exactly 320 ms long. The pitch of each word (and thus of the entire sentence) was flattened, and the intensity was normalized to 70 dB in Praat^50^. The words were concatenated into 80 semantically plausible and 24 semantically implausible 4-word sentences, which were further concatenated into trials each comprising 12 sentences (48 words). With 40 unique sentences per condition, we have developed a slightly smaller number of sentences than the sets of 50 used in other studies^14,30,48^ but note, importantly, that there were no sentence repetitions within a trial. The semantically implausible ‘outlier’ sentences were arranged by re-combining words from two semantically plausible sentences (e.g., Das Zelt lacht lahm; lit.: “The tent laughs lamely”) and were used as targets in the outlier detection task (see below). Each sentence was repeated 8 to 9 times within the same experimental block but never within the same trial. Each trial lasted for 15.36 s (12 sentences × 4 words × 320 ms; no pauses were introduced between words, phrases, and sentences), identical to the trials in Ding et al.^30^. For the two types of syntactic structure and for each type of prosodic contour used in the study (see below), we created 22 trials without any implausible outliers. In the experimental conditions that employed the outlier detection task, we added 8 additional trials with one implausible outlier sentence each, but these were not subjected to subsequent data analysis.

Syntactic structure of the sentences. Sentences followed one of the two types of syntactic structures. In the case of the 2 + 2 Syntax (40 sentences), sentences consisted of two syntactic phrases of equal length, comparable to Ding et al.^30^. The first phrase was a noun phrase (NP), consisting of a determiner and a noun, while the second one was a verb phrase (VP), most frequently comprised of a verb and an adverb (e.g., Der Tisch steht da; lit.: “The table stands there”, or “The table is over there”). In rare cases, the verb phrase (VP) consisted of a particle verb with the corresponding particle replacing the adverb (e.g., Mein Boot kippt um; English: “My boat tips over”). In the 1 + 3 Syntax (40 sentences), the first phrase in each sentence included a one-word NP (i.e., a name), and the second phrase was represented by a 3-word VP (typically, a verb and its complement, e.g., a verb + a determiner/preposition + a noun; e.g., Lars mag das Bild; English: “Lars likes the picture”; see Supplementary Materials A for the full list of sentences and additional details on their characteristics). The two types of syntactic structures were compared. Given that the current study used EEG (and not MEG, like the only frequency tagging study using 1 + 3 Syntax constructions with phrases of non-equal length), we first ran a control experiment in a separate group of participants to establish that the EEG effects for 1 + 3 groupings are analogous to the ones reported in Ding and coauthors’^14^ study (see Supplementary Materials B). Our results confirmed that 1 + 3 rhythm elicits an EEG spectrum similar to the MEG spectrum reported by Ding et al.^14^ and can, therefore, be contrasted with the 2 + 2 Syntax sentences in our main study. For both types of syntactic structure, the (acoustically unmarked) sentence boundary appeared once every 1.28 s (after four words) at a frequency of 1/1.28 (0.78) Hz (sentence frequency), and single words appeared every 320 ms (i.e., at a word frequency of 3.125 Hz). However, only in the 2 + 2 Syntax sentences (where the phrase boundary between the NP and the VP occurred after two words), syntactic phrases were isochronous and appeared at a constant frequency of 1.56 Hz (every 640 ms), that is, at ½ sentence frequency.

**Figure 2:**
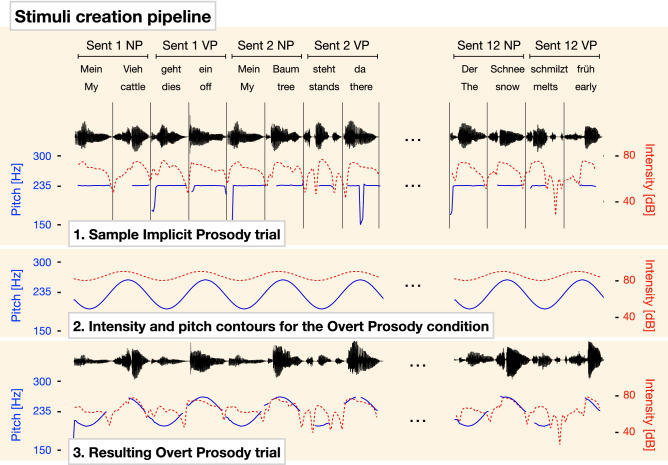
Stimulus design. Single words were synthesized and concatenated into trials (12 sentences each; sample sentences are taken from one of the 2 + 2 Syntax trials). In the Implicit Prosody condition (1), words were synthesized, and prosodic cues were neutralized: i.e., there were no pauses between words within trials, all words were 320 ms long, pitch was flattened, and sound intensity was constant across words. Artificial prosodic contours were then created using 1.56 Hz (½ sentence rate) sine waves (2) and imposed with neutralized prosody to create stimuli for the Overt Prosody condition. Pitch contour is depicted in blue (note that infrequent sudden drops of pitch values typically reflect unavailability of pitch information due to unvoiced phonemes), and sound intensity is represented by red lines. (3). Audio files for all stimuli are available online: https://osf.io/qzbne/.

Prosodic manipulations of the sentences and hypotheses. The sentences concatenated from words with neutralized prosody as described above constituted the Implicit Prosody condition (henceforth, ImplP) that was to be contrasted with the data from the Overt and Instructed Prosody conditions (henceforth, OvP and InstrP, respectively). As the general idea of our prosodic manipulation was to create prosodic patterns that would selectively support one syntactic structure (e.g., 2 + 2) while conflicting with the other one (e.g., 1 + 3), the most straightforward acoustic manipulation would have been to either insert pauses at a boundary position or increase the duration of pre-boundary syllables. This kind of prosodic manipulation changes the duration of pre-boundary words and has not only been found to be the most reliable boundary marker in natural speech, but has also been successfully used in previous studies to create cooperative and conflicting syntax-prosody pairings^46^, including in EEG studies^47,51,52^. However, in a frequency tagging study that crucially depends on the invariable duration of all syllables, phrases, and sentences, durational manipulations are not an option. Instead, we manipulated pitch and intensity, two prosodic dimensions that also contribute to prosodic boundary marking^53–56^. To this end, we synthesized artificial pitch and sound intensity contours in Matlab R2019a. The resulting artificial prosodic contours were then imposed onto the sentences of the ImplP condition (using Praat^50^), thereby creating the OvP condition (see Fig. 2).

In the OvP condition, the maxima of sound intensity and pitch were placed on Words 2 and 4 (hence, W24 contour). That is, the fluctuations of pitch and intensity appeared at the phrasal (½ sentence) frequency (1.56 Hz). Avoiding strategic carry-over effects across sentence conditions, other prosodic contours were tested in alternation with the W24 contour, but these are irrelevant for our present findings and will be reported elsewhere. We imposed the W24 contour onto all experimental sentences of both 2 + 2 and 1 + 3 Syntax structures. We consider the W24 prosodic contour to be optimally aligned with the syntactic phrasing of the 2 + 2 Syntax sentences: using prosodic phrasing cues suitable for a frequency tagging paradigm (i.e., avoiding changes in lengthening), we created a simplified model of a prosodic 2 + 2 pattern that was expected to support the phrasing of two isochronous syntactic phrases.

An important consequence of this type of prosodic manipulation is that, while it is expected to support a certain syntactic type of phrasing (i.e., the 2 + 2 Syntactic structure), it could potentially interfere with the default 2 + 2 prosodic phrasing that listeners (and silent readers) may implicitly apply to any kind of syntactic structure compatible with this prosodic phrasing. To the extent that default prosodic phrasing already depends on a specific realization of prosodic boundaries (e.g., the mental postulation of a break^20,23,29^), a different prosodic contour that does not share the same prosodic (or acoustic) features is likely to cause a conflict, even if both prosodic contours support the same 2 + 2 phrasing pattern. To put it differently, the human brain may be able to combine a given syntactic structure with different types of prosodic realizations, but it is unlikely that it can handle two distinct prosodic realizations for the same sentence in parallel. For these reasons, we predicted stronger interference effects for the introduction of artificial prosodic contours (in OvP and InstP conditions) whenever the participants’ default phrasing (in the ImplP condition) was not limited to syntactic processing but already relied on prosodic processing (and a specific ‘self-generated’ prosodic contour that was different from our artificial contour). Clearly, such a scenario is more likely for 1 + 3 than 2 + 2 syntactic structures.

For the W24 contour used in our prosodic manipulation, we predicted an enhancement of ½ sentence rate EEG responses in the case of syntax-prosody alignment (2 + 2 Syntax) and expected this effect to be stronger than any analogous effect in the 1 + 3 Syntax sentences, for two very different reasons. *First*, because the 1 + 3 Syntax sentences with the W24 prosodic contour may present the case of a potential misalignment between syntactic and prosodic grouping. In this case, we might expect to see reduced brain activity at the frequency of the sentence due to participants’ hindered ability to phrase sentences in their preferred (spontaneous) way. However, as explained above, this misalignment argument relies on the assumptions that (a) frequency peaks related to top-down structural tracking are exclusively driven by syntactic phrasing and that (b) syntactic and prosodic phrasing have to be aligned. In our opinion, both assumptions are likely to be wrong: after all, we have argued that implicit prosody may indeed contribute to top-down frequency peaks and that a 2 + 2 prosody is perfectly compatible with our present 1 + 3 Syntax sentences (but not with those used by Ding et al.^14^). We assume, therefore, that the *second* factor that can shape the differential effect of prosodic grouping on the processing of sentences with different syntactic structures is in fact rather the interplay between the default, “spontaneous” prosodic contour applied to the sentences and the artificial prosodic contour we have created. While the W24 prosodic contour (as a specific acoustic realization of a 2 + 2 prosodic phrasing) should—in principle—be as compatible with our 1 + 3 Syntax sentences as with our 2 + 2 Syntax sentences, we expect a higher degree of interference in 1 + 3 sentences due to the different nature of the ½ sentence peak. In 1 + 3 Syntax sentences, unlike in 2 + 2 sentences, only spontaneous implicit prosodic phrasing (but not syntactic phrasing) could elicit a ½ sentence peak (in the ImplP condition), and the underlying implicit prosodic contour is more than likely to be incompatible with the artificial prosodic contour imposed by the W24 manipulation. The conflict between two competing prosodic contours is predicted to decrease rather than enhance the ½ sentence peak amplitude.

In sum, we predicted that our prosodic manipulations would affect the two sentence types differently and result in a syntax by prosody interaction for the amplitudes of the relevant frequency peaks. If so, a purely syntactic account for those peaks would be insufficient.

We tested all experimental sentences for intelligibility in the Overt Prosody condition in 7 pilot participants who did not subsequently participate in the EEG recordings, while the Implicit Prosody intelligibility data were collected from every participant at the beginning of the main EEG experiment (including 11 participants who did not go through the full experiment; see Supplementary Materials A).

In the InstrP condition, the sentences used in the Implicit Prosody condition were presented again (in a different order) but each of them was preceded by a trial of OvP sentences (see Procedure below). Participants were asked to silently apply the same prosodic contour they had just heard in the OvP condition while listening to the sentences. Contrasting the OvP and the InstrP conditions allowed us to identify the role of ‘overt’ prosody (acoustically realized in OvP sentences) and imagined, instructed (‘covert’) prosody (in the InstrP condition).

## Procedure

Every participant visited the lab for 5–6 h, including a 3.5–4.0 h period of EEG recording involving multiple experiment parts, with several breaks throughout the EEG session. During the EEG cap setup, participants filled out behavioural questionnaires. After that, they performed a stimulus familiarization task. The experimenter explained to the participants that the stimuli were synthesized and the speech rate was relatively high, which is why some of the sentences might possibly be difficult to understand right away. To avoid any comprehension problems during the EEG study, participants had an opportunity to read through the full list of sentences (including the semantic outliers) prior to the experiment and then performed a computerized sentence intelligibility task (note that exposing participants to the stimuli prior to the main experiment was done in previous research as well^57^). In this task, participants listened to every sentence (with a maximum of two replays) and typed in what they heard. Using this task, we were able to verify that all participants understood the vast majority of the sentences: on average, they correctly typed in 100 out of 104 sentences (for results, see Supplementary Materials A). Following the behavioural task, the main series of EEG experiments started.

**Figure 3:**
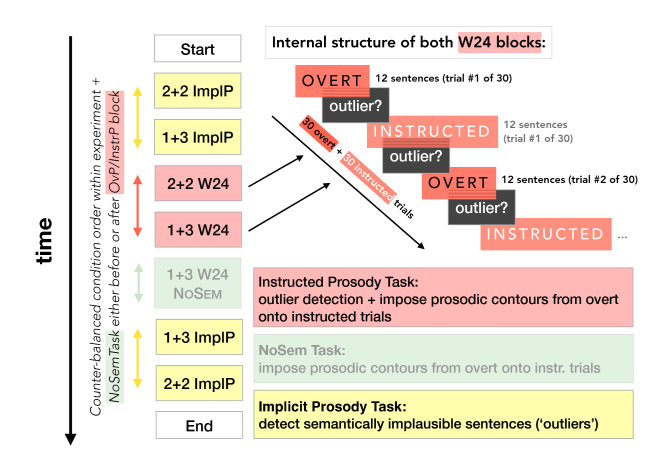
Experimental procedure. Left: order of the experimental blocks. In yellow—the Implicit Prosody (ImplP) conditions with the corresponding 1 + 3 and 2 + 2 Syntax blocks. In pink– both Overt Prosody (OvP) and Instructed Prosody (InstrP) conditions. Note that participants were presented with an additional, No Semantic Task condition (faded green block), which is largely beyond the scope of the current experiment. The details regarding this part of the study are briefly summarized in Supplementary Materials C. The No Semantic Task condition was always represented by one block composed of sentences with one of the syntactic structures. Right (top): scheme of experimental flow within one sample block of combined OvP and InstrP trials (for example, 2 + 2 W24 = 2 + 2 Syntax sentences with W24 prosodic contour). Starting with an OvP trial (12 sentences), it continues with an outlier detection prompt, and then with the InstrP trial, during which participants listen to sentences with neutralized prosody while imposing onto them the intonational contour they attended to in the preceding OvP trial. In the bottom right corner is the list of tasks used in the corresponding experimental conditions.

Every participant started with listening to the Implicit Prosody trials that served to establish a baseline for their syntactic processing of 1 + 3 and 2 + 2 Syntax sentences and, potentially, for their default implicit prosodic phrasing as well. This condition is comparable to that in Ding et al.^30^. Next, participants were presented with OvP and InstrP conditions. At the end of the study, we presented the ImplP condition again (with a randomized trial order different from the one at the beginning of the experiment) to control for possible sentence familiarity differences between the ImplP and other conditions, as well as changes in participants’ fatigue over the course of the study. Ultimately, the data from the two runs of the ImplP condition were averaged after ensuring the main patterns were unaffected by whether the data were recorded at the beginning or at the end of the experiment (see Results). OvP and InstrP trials were presented in blocks containing trials with the same prosodic and syntactic structure. The order of 2 + 2 and 1 + 3 Syntax blocks was counter-balanced across participants within each of the conditions (Fig. 3). Overall, the sequence of blocks was chosen to minimize the influence of any condition on processing strategies in the respective next block.

In the Implicit Prosody condition, participants listened to 30 trials containing the sentences with neutralized prosody (22 without and 8 with semantic outliers). At the end of each trial (i.e., after listening to 12 consecutive sentences), they had to indicate via button press if that trial contained a sentence that did not make sense (an ‘outlier’), or if all sentences were plausible. Trials consisting of the sentences with the same syntactic structure (1 + 3 or 2 + 2 Syntax) formed a block, with the order of blocks being counter-balanced across participants.

In the Overt and Instructed Prosody conditions, trials were presented in pairs (Fig. 3). Participants first listened to a trial of 12 sentences, all of which had identical syntactic structures and the same overt prosodic contour (e.g., 1 + 3 Syntax with W24 contour). This Overt Prosody trial was immediately followed by a second Instructed Prosody trial of 12 sentences, which still had the same syntactic structure as before (here: 1 + 3 Syntax) but lacked any prosodic contour (similar to the Implicit Prosody condition). During this second trial, participants were instructed to silently ‘imagine’ the same prosodic pattern they had just heard during the overt prosody trial (here: W24). In other words, participants were asked to process the sentences while imposing a covert prosodic contour. This Instructed Prosody trial inherited its prosodic characterization from the preceding overt prosody trial (i.e., 2 + 2 syntax with covert, instructed W24 contour). Comparing the EEG signals of these trials to the Implicit Prosody conditions should reveal the contribution of both overt and covert prosody to the elicitation of power peaks. After each trial (with overt or covert prosody) participants had to indicate by button press if that trial contained a semantic outlier sentence or not.

### EEG recording and processing

EEG data were recorded at a 500 Hz sampling rate using 64 cap-mounted electrodes (extended International 10–20 electrode organization System, Jasper, 1958; Waveguard™ original ANT Neuro EEG system), referenced online to the right mastoid. Matlab and EEGLAB (version 14_1_0b^58^) were used for EEG data preprocessing (the code is available online: https://osf.io/qzbne/). Offline, we re-referenced the data to linked mastoids, removed bridged electrodes (the values were interpolated from the neighbouring electrodes after extracting epochs from the data), and performed resampling of the continuous datasets to 250 Hz. We filtered the data separately with a low- (20 Hz cut-off, filter order = 152) and a high-pass (0.2 Hz cut-off, filter-order = 2266) FIR filter using the Kaiser window (β = 5.65326). We removed eye movement artifacts using Independent Component Analysis^59^ (ICA) run on the strongly high-pass filtered copies of the original datasets (1 Hz cut-off, filter order = 454). Note that we used these datasets for ICA decomposition only, for which we cut them into dummy epochs that underwent automatic artifact removal. Epochs were removed whenever the EEG at any time point (1) exceeded the threshold of ± 400 μV, (2) deviated at any of the electrodes from the mean amplitude at that electrode by 2 SDs, or (3) deviated at any of the electrodes from the mean of activity at all electrodes by 6 SDs. We copied the results of the ICA decomposition back onto the continuous data from which the activity of the components accounting for eye movements was then subtracted.

For frequency tagging analysis, the data were cut into 14.08-s long epochs time-locked to the beginning of the second (rather than the first) sentence in each trial to avoid transient noise associated with the processing of the beginning of a given trial. Epochs containing signal crossing the ± 40 dB threshold in the 0–4 Hz frequency range were removed. The mean of each epoch was subtracted from each data point in it, after which EEG was averaged across trials, resulting in one average for each participant, electrode, and experimental condition. We calculated the evoked power assessed using the fast Fourier transform (FFT) of time-domain EEG responses averaged across trials (i.e., the FFT of the ERP, representative of the power of brain activity synchronized with the speech input). The resulting resolution of the frequency data was 0.071 Hz.

Due to the uneven distribution of noise across the frequency domain, the evoked power was further normalized by dividing the power at every frequency bin value in the spectrum by the mean of the response power at 14 neighbouring bins comprising 0.5 Hz prior as well as 0.5 Hz following that frequency bin (7 bins of 0.071 Hz on each side of the target one). The resulting data can be seen as the signal-to-noise (SNR) ratio of the EEG power across the frequency spectrum.

### Statistical analysis

Behavioural binomial generalized mixed-effects models (lme4 package in R^60^) were built following forward-directed model comparison based on the Akaike Information Criterion (AIC) (in the case of the EEG data, this was done using the buildmer package^61^). Relevant details of the final models are presented in the Results section. For the analysis of behavioural responses in the ImplP, OvP, and InstrP conditions, we fitted two generalized binomial models. The first one tackled the effect of Prosody (OvP, InstrP, or ImplP). The fixed effects tested for inclusion into this model were Prosody, Syntax (1 + 3 vs. 2 + 2), and Item Type (Correct vs. Outlier), as well as all possible interactions between them while the model was converging. Random intercepts for each participant and item were included by default, prior to model comparison. All fixed effects included in the best model were then tried as random slopes when appropriate. The second model tested the potential effect of alertness and familiarity on semantic plausibility task responses. The build-up procedure was similar to the one described above, but the only fixed effects tested for inclusion were Experiment Part (Beginning vs. End), Syntax, and Item Type. Additionally, d-prime values were calculated to form one of the predictors of the EEG data (as behavioural task responses have been shown to correlate with sentence-level EEG effects by Ding and coauthors^30^).

In the analysis of EEG data, first, normalized EEG power at the sentence (0.78 Hz) and the ½ sentence (1.56 Hz) frequencies was tested using bias-corrected and accelerated bootstrap tests (as implemented in the wBoot R package^62^) against the normalized power at the neighbouring frequencies (7 frequency bins on each side from the target frequency bin). This was done separately for each experimental block (see Fig. 3). All *p* values were Bonferroni-corrected for multiple comparisons. We extended this analysis by directly and systematically studying the normalized EEG power at the target frequencies, minus the noise at the 14 surrounding frequency bins, across our main experimental conditions using two generalized linear mixed-effects models (one for the effects at the ½ sentence rate, another one for the effects at the sentence rate). The models were fitted with Gamma distribution and an identity link function. To allow for the use of the Gamma distribution, we added a minimal constant to the dependent variable shifting the values into the (strictly) positive range. Forward-directed comparison was performed analogous to the process implemented during behavioural data analysis, with all models including random intercepts by participant. The effects tested for inclusion into the models were Prosody, Syntax, Anteriority (Frontal vs. Central vs. Posterior channels); Laterality (Left vs. Medial vs. Right channels), d-prime values, and all possible interactions between them. Potential side effects of familiarity and alertness of the participants were investigated by building an additional model where only the data from the ImplP conditions were used. In this model, the effects tested for inclusion were: Experiment Part (Beginning vs. End), Syntax, Frequency, Anteriority, Laterality, Frequency, the d-prime values, and all possible interactions between them.

All linear models were visually checked for normality and homoscedasticity of the residuals. The absence of multicollinearity was ensured based on the condition number^63^ and the variance inflation factor^64^. The post-hoc analysis of interactions was performed by comparing the least-squares means and their standard errors (as implemented in the lsmeans package^65^).

We additionally studied the relationships between our EEG effects (i.e., the ½ sentence and the sentence rate EEG responses within and between different conditions) using Pearson’s correlation to investigate our interpretations regarding the (dis)similarity of some findings (see Results).

## Results

### Performance on the behavioural task

On average, participants were 72.2% accurate in assessing the semantic acceptability of the sentences (comparable to results of Ding et al.^30^; see percentages per condition in Supplementary Figure S2). Performance was higher on trials without outliers than on those with semantically implausible sentences (β = − 0.67, SE = 0.037, *p* < 0.001). Within the group of semantically plausible sentences, response accuracy on the semantic plausibility task (identification of outliers as outliers and correct sentences as not being outliers) was slightly higher for the 1 + 3 Syntax compared to the 2 + 2 Syntax conditions (Item Type × Syntax: β = − 0.141, SE = 0.037, *p* < 0.001; Correct items: 1 + 3 – 2 + 2 Syntax: β = 0.312, SE = 0.083, *p* = 0.001). Participants performed slightly better at assessing acceptability of trials with overt or instructed prosody compared to trials in the ImplP conditions (ImplP–InstrP: β = − 0.952, SE = 0.083, *p* < 0.001; ImplP–OvP: β = − 0.883, SE = 0.083, *p* < 0.001). In addition, we found that acceptability of trials without outliers within the ImplP condition improved towards the end of the study (β = − 0.411, SE = 0.106, *p* < 0.001). Syntactic structure of sentences did not significantly improve the ImplP model and was not included as a fixed effect.

### EEG results

#### Implicit prosody condition

**Figure 4:**
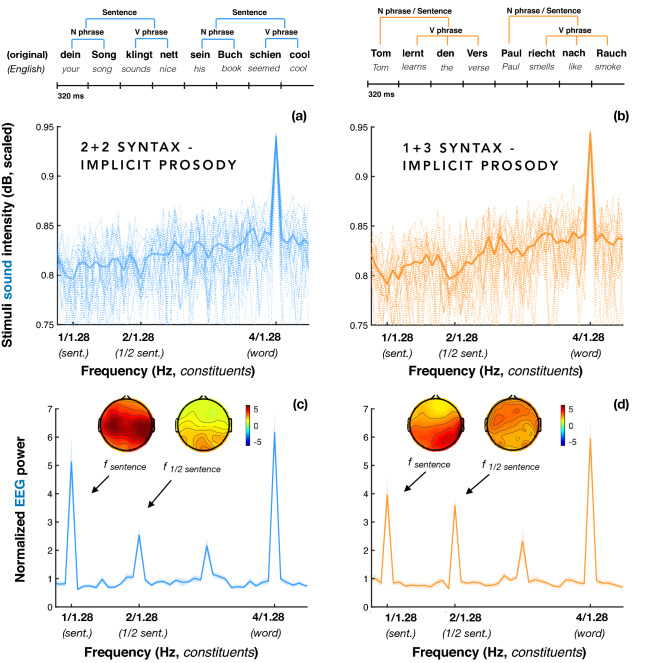
Spectral results: Implicit Prosody condition. Left panel (from top to bottom): sample sentences with the 2 + 2 syntactic structure, spectrum of sound intensity envelopes of the stimuli [(**a)** thin dotted lines represent single trials, bold line depicts the average across all trials], the EEG power spectrum recorded while participants were listening to the 2 + 2 Syntax sentences (**c**). Right panel (**b**,**d**): same for 1 + 3 Syntax. Note that the main syntactic boundary in the 1 + 3 Syntax condition (i.e., the one between first and second words) is not reflected in the spectrum, because the phrases forming the 1 + 3 Syntax condition are non-isochronous. The lines in the spectrum plots reflect group averages, with the shaded area depicting standard errors of the mean. Scalp maps depict scalp distribution of the EEG signal at the sentence and the ½ sentence frequencies (quantified as distance to the signal at surrounding frequencies). The peak at 3/1.28 Hz (the rate at which no syntactic or prosodic cues were modulated), represents a harmonic of the sentence frequency, similar to the 3 Hz response in the 1 + 3 Syntax condition in Ding and colleagues’ original study (2016).

EEG power at word, sentence, and ½ sentence frequencies in 1 + 3 and 2 + 2 Syntax was significantly larger than noise (all *p* < 0.001; Fig. 4c,d), a pattern strikingly different from the one observed in the spectrum of the corresponding sound intensity envelopes (Fig. 4a,b); in what follows we will focus on the relevant sentence and ½ sentence peaks. The sentence-level effects were in line with our predictions for both types of sentences but they did not differentiate between the theoretical accounts tested (see Fig. 1). At the same time, according to the syntactic account, the peak in the EEG spectrum at the ½ sentence frequency would be expected only in the case of the 2 + 2 Syntax (corresponding to the phrase frequency; see Fig. 1a,b). However, this ½ sentence EEG peak was significant in the 1 + 3 Syntax condition as well, and was even found to be somewhat larger than in the 2 + 2 condition (β = 1.063, SE = 0 0.203, *p* < 0.001). The elicitation of this peak was also replicated in a larger group of participants (N = 36) who only took part in the first run of the Implicit Prosody condition. We therefore conjecture that at least the ½ sentence EEG peak in the 1 + 3 Syntax condition was likely induced by mechanisms other than syntactic processing, and specifically, in our view, by participants having placed a covert prosodic boundary in the middle of the 1 + 3 Syntax sentences. We further tested this hypothesis by investigating the correlations between the different EEG peaks in the ImplP condition and by comparing their scalp distributions. We hypothesized that if the ½ sentence EEG peak in the 1 + 3 Syntax condition is driven exclusively by prosody, (1) its size would vary differently across participants than the other EEG peaks that are at least partly influenced by syntactic processing, and (2) this modulation might have a distinct scalp distribution.

Indeed, we found no correlation between the ½ sentence peaks in the two types of syntactic constructions (r^2^ = 0.033, *p* = 0.872), while the sentence peaks were indeed positively correlated (r^2^ = 0.479, *p* = 0.013). We have also seen that across all prosodic conditions, the ½ sentence peak in 1 + 3 Syntax sentences has a less posterior scalp distribution than the one in the 2 + 2 Syntax sentences (see the OvP and InstrP results subsection below). Taken together, these findings suggest that the ½ sentence peaks in the two syntactic conditions were of different nature.

As the ImplP condition was run twice (once at the beginning and once at the end of the experiment), we compared these measures to estimate the effects of familiarity and alertness on results. While the EEG responses at the sentence and the ½ sentence frequencies were reduced at the end compared to the beginning of the experiment (β = 0.944, SE = 0.071, *p* < 0.001), this effect was not specific to the type of syntactic structure (and can thus not account for the differential effects in 1 + 3 versus 2 + 2 sentences we will turn to next). To minimize the potential impact of order or fatigue effects on contrasts between conditions, all comparisons of the ImplP condition with other conditions used an average of all ImplP data (from early and late recordings).

#### Overt and Instructed Prosody conditions

**Figure 5:**
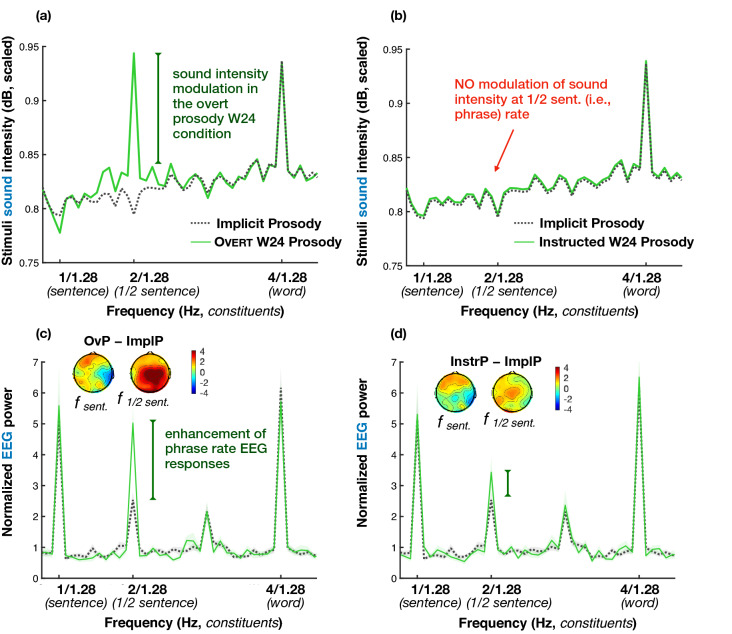
EEG results for the 2 + 2 Syntax sentences with the prosodic contour aligning with their syntactic structure plotted against the data from the same 2 + 2 Syntax sentences in the Implicit Prosody condition (ImplP, dotted grey lines): Overt Prosody (OvP, left column) and Instructed Prosody (InstrP, right column) conditions. The top row (**a**,**b**) represents the spectrum of sound intensity envelopes of the spoken sentence materials, the bottom row depicts EEG power in listeners (**c**,**d**). The lines in the spectrum plots reflect group averages, with the shaded area depicting standard errors of the mean. Scalp maps show the scalp distribution of the difference between EEG peaks (calculated as distance from the peak value to surrounding noise) for OvP minus ImplP in (**c**) and for InstrP minus ImplP in (**d**) (separately for sentence and ½ sentence frequencies). Key effects are marked with vertical bars: when a prosodic W24 contour is applied to the 2 + 2 sentences in which it aligns with syntactic phrasing (whether overtly or covertly), the EEG responses were enhanced compared to the ImplP condition.

The EEG data from the OvP and InstrP conditions are depicted in Fig. 5 (2 + 2 Syntax sentences) and 6 (1 + 3 Syntax sentences). As in the case of the ImplP sentences, spectral amplitude at sentence and ½ sentence frequencies in every experimental condition in the Prosody experiment was significantly larger than noise (all *p* values < 0.001). Across the OvP, InstrP, and ImplP conditions, we found differences between scalp regions (i.e., the effect of Anteriority). The sentence frequency responses, independent of the experimental condition, had a centro-posterior distribution (frontal-central: β = − 0.641, SE = 0.151, *p* < 0.001, frontal-posterior: β = − 0.374, SE = 0.143, *p* = 0.024). A somewhat more posterior distribution of EEG power peaks was seen in the 2 + 2 Syntax condition at the ½ sentence frequency (frontal-central: β = − 0.548, SE = 0.183, *p* = 0.033, frontal-posterior: β = − 1.51, SE = 0.224, *p* < 0.001, central-posterior: β = − 0.96, SE = 0.233, *p* < 0.001). The EEG responses at the ½ sentence frequency in 1 + 3 Syntax sentences were more broadly distributed (no interaction with Anteriority) and were smaller than those in 2 + 2 Syntax when contrasted in the posterior region (β = − 0.895, SE = 0.241, *p* = 0.003). Note that no interactions between Contour and Anteriority were observed. We next analyzed the results by comparing peaks across experimental conditions to test our hypothesis about (i) the effects of overt and covert prosody as well as (ii) the syntax-prosody alignment on the EEG responses at frequencies of syntactic constituents. We found a significant effect of Prosodic Contour × Syntax at both ½ sentence (β = 0.603, SE = 0.086, *p* < 0.001) and sentence frequencies (β = 0.293, SE = 0.101, *p* = 0.004). That is, superimposing a W24 prosodic contour had different effects on EEG spectral peaks elicited by the 2 + 2 compared to the 1 + 3 Syntax sentences.

**Figure 6:**
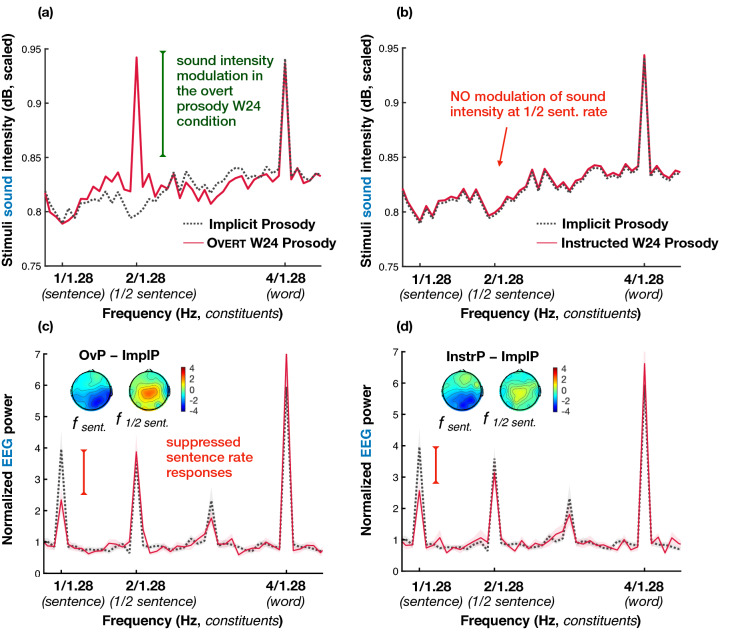
EEG results for the 1 + 3 Syntax sentences with the prosodic contour misaligned with their syntactic structure (thin red lines) plotted against the data from the same 1 + 3 Syntax sentences in the Implicit Prosody condition (ImplP, dotted grey lines): both Overt Prosody (OvP, left column) and Instructed Prosody (InstrP, right column) conditions. The top row (**a**,**b**) represents the spectrum of sound intensity envelopes of the spoken sentence materials, the bottom row depicts EEG power in listeners (**c**,**d**). The lines in the spectrum plots reflect group averages, with the shaded area depicting standard errors of the mean. Scalp maps show the scalp distribution of the difference between EEG peaks (calculated as distance from the peak value to surrounding noise) for OvP minus ImplP in (**c**) and for InstrP minus ImplP in (**d**) (separately for sentence and ½ sentence frequencies). Prominent significant effects are marked with vertical bars: when a prosodic W24 contour not aligning with the 1 + 3 syntactic structure was overtly or covertly applied to the sentences, EEG responses were diminished compared to the ImplP condition.

Figure 5 illustrates the comparison between the ImplP and the W24 prosodic contour in the case of syntax-prosody alignment—i.e., in 2 + 2 sentences, depicting first the sound intensity spectrum of the speech signals (Fig. 5a,b) and then the spectra for EEG signal (Fig. 5c,d). When participants listened to 2 + 2 Syntax sentences with an overt W24 prosodic contour (left panel), EEG power at the ½ sentence frequency was higher compared to the ImplP and InstrP condition (ImplP–OvP: β = − 1.883, SE = 0.235, *p* < 0.001; InstrP–OvP: β = − 1.339, SE = 0.246, *p* < 0.001).

Given the substantial impact the W24 pattern had on the ½ sentence peak in terms of the acoustic spectrum (Fig. 5a), it could be argued that the corresponding EEG changes at this frequency (Fig. 5c) might, in principle, be driven by bottom-up acoustic changes. Crucially, however, the instructed prosody condition resulted in similar, though smaller EEG changes as the overt prosody condition. That is, when participants were presented with the exact same (prosodically neutralized) 2 + 2 sentences as in the ImplP condition—but were asked to imagine them with the W24 prosodic contour (in absence of any overt prosodic cues in the speech signal, see Fig. 5b)—, we once again observed very similar EEG effects (Fig. 5d). The ½ sentence EEG power was significantly larger for the sentences with the covert W24 prosodic contour compared to the ImplP condition (β = − 0.544; SE = 0.191; *p* = 0.049), though the effect was smaller than that of OvP.

In contrast to the 2 + 2 Syntax, when 1 + 3 Syntax sentences (Fig. 6) were presented with the overt W24 contour (i.e., the prosodic contour was less aligned with the 1 + 3 Syntax structure), this combination elicited EEG power that was significantly smaller than in the ImplP condition at the sentence rate (ImplP–OvP: β = 0.922, SE = 0.201, *p* < 0.001). Importantly, an analogous suppression of EEG responses was observed when the W24 prosodic contour was applied to the sentences covertly, or imagined by the participants (ImplP–InstrP: sentence frequency: β = 0.849, SE = 0.202, *p* < 0.001; ½ sentence frequency: β = 0.777, SE = 0.210, *p* = 0.003; see Fig. 6). When OvP 1 + 3 and 2 + 2 Syntax sentences were compared directly, we saw that the 2 + 2 Syntax sentences had larger EEG power at both ½ sentence and sentence frequencies (1 + 3–2 + 2 Syntax: β = − 1.235, SE = 0.251, *p* < 0.001 and β = − 1.567, SE = 0.250, *p* < 0.001, respectively). In the InstrP condition, this difference reached significance only for the sentence frequency (β = − 1.55, SE = 0.245, *p* < 0.001), however, the larger ½ sentence peak amplitude for 1 + 3 we saw in the ImplP condition is not present either.

**Figure 7:**
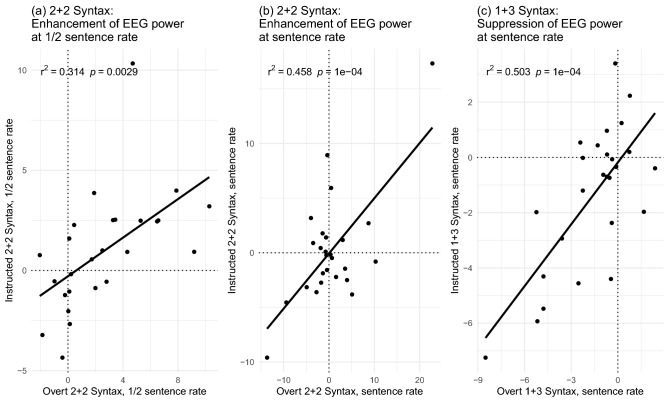
Correlations between effects of overt and instructed W24 prosodic contour strongly aligned with the 2 + 2 Syntax and less aligned with the 1 + 3 Syntax sentences. The correlations between enhancement of EEG responses in InstrP and OvP conditions contrasted with the Implicit Prosody EEG spectrum peaks at the (**a**) ½ sentence rate and (**b**) sentence rate in the case of strong syntax-prosody alignment (2 + 2 Syntax W24 prosodic contour). (**c**) In the case of weaker syntax-prosody alignment (1 + 3 Syntax W24 prosodic contour), the sentence rate suppression of EEG responses in OvP and InstrP conditions were correlated as well. The values represent differences in normalized EEG power (in arbitrary units, scaled) between Implicit Prosody and either Overt or Instructed Prosody (OvP-ImplP and InstrP-ImplP) experiments at corresponding frequencies. That is, at zero on either of the axes, there is no difference between ImplP and the prosody condition; negative values reflect reduction of the EEG spectrum peak by the application of the W24 prosodic contour; positive values reflect enhancement of the EEG spectrum peak in the W24 compared to the ImplP condition.

The differential effect that the W24 prosodic contour had on 1 + 3 versus 2 + 2 Syntax sentences was as predicted. When prosody is fully aligned with the syntactic structure, cortical responses at the syntactic constituent rates are enhanced compared to sentences with neutralized prosody. When, on the other hand, the prosodic contour does not align with syntactic phrasing and/or conflicts with participants’ spontaneous (default) prosodic contour, we see reduced cortical responses compared to sentences with neutralized prosodic cues. Moreover, the effects of overt and instructed (covert) W24 prosody are strikingly similar. This extends to the fact that participants with a larger enhancement of ½ sentence and sentence rate responses by the W24 contour overtly applied to 2 + 2 Syntax sentences were also the ones with the larger effects in the InstrP condition. Similarly, larger suppression of the sentence-level responses by overt W24 prosody in the 1 + 3 Syntax sentences is associated with larger suppression for covert W24 prosody in the InstrP trials (Fig. 7).

## Discussion

In naturalistic running speech, we hear syntactic groupings typically only if they are expressed prosodically, e.g., by acoustic boundary markers such as breaks, syllable lengthening, or pitch changes. However, prosodic groupings are not always driven by syntactic structure; prosodic boundaries can also be motivated by non-syntactic principles, such as the ‘same sister’ constraint that leads to prosodic phrases with equal numbers of syllables, independent of syntactic constituents^23,31,66^, or by acoustically longer syntactic phrases that do not change the syntactic structure^29^. Because syntactic and prosodic processing are distinct but closely associated, accurate understanding of sentence processing mechanisms requires careful consideration of both prosody and syntax and their interaction. Even when a speech signal is lacking overt prosodic cues (such as in Ding et al.^14^), language users can and do activate implicit, or covert, prosodic contours during processing^23,24,67^. Since both overt and implicit rhythmic groupings are known to entrain brain activity and result in corresponding EEG and MEG power increases at the rhythm’s frequency^68,69^, it seems essential to rule out the contribution of prosodic rhythms before EEG and MEG power peaks can be attributed to other cognitive domains, including hierarchical syntactic processing. Using three experimental conditions, we studied how neural responses to linguistic phrases and sentences are modulated by both overt and covert prosody. We used the frequency tagging approach, a method recently applied to the study of sentence processing. Although results from recent frequency tagging studies^14,30^ have been interpreted as evidence for cortical tracking of hierarchical syntactic structure, our data show that other top-down mechanisms, and covert prosodic phrasing in particular, must be considered to account for these effects. We showed that low frequency cortical activity tracks both overt and covert prosodic changes, and this tracking interplays with syntactic processing.

We approached the investigation of prosodic processing in several steps. First, we conducted an EEG pilot study testing if non-word groupings based exclusively on pitch manipulations (2 + 2: low-low-high-high versus 1 + 3: low-high-high-high) would replicate Ding et al.’s MEG findings for 2 + 2 and 1 + 3 syntactic structures. As expected, we found a significant EEG power peak corresponding to a 2-word grouping (½ sequence peak in Supplementary Materials B) only for the 2 + 2 but not for 1 + 3 grouping condition. These data demonstrate that the human brain tracks acoustically marked rhythmic groups of quasi-linguistic word materials, and that EEG measures capture this process.

Next, we studied two types of real sentences with either 2 + 2 or 1 + 3 syntactic structure in which overt prosodic cues were neutralized (Implicit Prosody condition), much like in Ding et al.^14^. However, unlike Ding et al.^14^, we unconfounded syntactic and possible covert prosodic structures, such that 1 + 3 sentences were compatible with a prosodic boundary in mid-sentence position (2 + 2 prosody). EEG power measurements showed strong peaks at sentence and ½ sentence (phrasal) frequencies for both sentence types. A purely syntactic account can only explain the ½ sentence peak for the 2 + 2, but not for the 1 + 3 syntactic structure: indeed, Ding et al.^14^ used the absence of this peak in their 1 + 3 structures as evidence for a structural account reflecting syntactic phrase processing. Our tentative interpretation is that the ½ sentence peak in the 2 + 2 condition reflects some extent of top-down syntactic processing (in line with previous studies), even though it may be partly mediated by implicit prosody, whereas the corresponding peak in 1 + 3 sentences emerges exclusively from listeners’ spontaneous implicit prosodic phrasing. This qualitative difference between sentence conditions was further supported by both (a) a significantly more posterior scalp distribution of the ½ sentence peak in 2 + 2 than 1 + 3 sentences, and (b) the lack of a significant correlation between the peak amplitudes in the two conditions. The former finding suggests that cortical tracking of prosodic phrases (in 1 + 3 sentences) is associated with more frontal activity at the scalp than tracking of syntactic phrases (this interpretation is also in line with the task effects reported in the No Semantic Task condition; see Supplementary Materials C). More anterior frequency peaks for prosodic than syntactic phrasing is consistent with previous fMRI work by Meyer et al.^44^, which identified frontal brain circuits for both prosodic processing (in the left hemisphere) and prosody production (in the right hemisphere; which may play a role in implicit prosody). These differences in peak topography would not be expected if the peaks reflected the same cognitive processes in both conditions. Keeping in mind the absence of the ½ sequence peak in our pilot non-word experiment, it is implausible that a harmonic account could explain the ½ sentence peak. Implicit prosody can, moreover, arguably account for some aspects of the recent frequency tagging results investigating the relationship between harmonic structure and sentence grammaticality in the German language^70^. Similarly, our prosodic account of the ½ sentence peak in 1 + 3 sentences is compatible with the results of a recent study in Russian showing this peak in both 1 + 3 and 2 + 2 syntactic structures while elegantly controlling for lexico-semantic features^48^. In addition to these expected differences in scalp distribution, the ½ sentence peak in 1 + 3 sentences was also found to be substantially larger than in the 2 + 2 condition, which was not predicted. We speculate that this difference in peak magnitude may point to generally stronger neural activity in circuits involved in prosodic (as compared to syntactic) phrasing. Consistent with this suggestion, recall that strong electrophysiological effects for rhythmic entrainment in both the auditory and the visual domain, in both humans and non-human mammals, belong to the earliest reliable electrophysiological findings^71–74^, whereas neurolinguistic research has generally struggled to identify reliable brain markers for hierarchical syntactic processing. Overall, our data suggest both a contribution of top-down syntactic processing (in 2 + 2 sentences) and top-down prosodic phrasing (in 1 + 3 sentences).

In contrast to the ½ sentence peaks, the power peaks at full sentence frequency were significantly correlated between syntactic structures in terms of amplitude, and their scalp distributions were found to be strikingly similar as well, suggesting similar underlying cognitive processes across 2 + 2 and 1 + 3 sentence structures. In absence of any acoustic markers for sentence boundaries in the speech signal, the power peaks at sentence frequency must reflect the top-down integration of four words into a coherent sentence representation. Whether this cognitive process is primarily syntactic in nature (as previously suggested by Ding et al.^14^) or stems from semantic processing^48,75^ (be that lexical semantics or thematic role processing) or combinatorics^22^ is not yet fully understood. Recent work by Lu et al.^76^ suggests that the relevant cognitive processes operate at the phrasal or sentential level and can not be accounted for by semantic processing at the word level. Importantly, covert prosodic processing can be driven by both semantic and syntactic cues^20,31^, meaning the sentence-level peak may be mediated by prosody as well.

Several lines of previous research support the idea that sentence prosody is tracked by slow neural activity. Studies of the Closure Positive Shift^24^ (CPS) showed that both overt and covert prosodic boundary processing is reflected in a slow event-related potential (ERP) component lasting for up to 400–500 ms. At the same time, we know that the time window corresponding to the rate of cortical delta oscillations (1–4 Hz) encompasses the length of acoustic chunks that can be efficiently processed in behavioural paradigms^77,78^. The link between the delta oscillatory range and behavioural data on the delta oscillatory range is supported by Meyer et al.^79^ reporting that delta oscillation phase changed at phrase boundary positions, whether phrase boundaries were driven by prosodic change or by parsing choices (in the absence of overt prosodic cues). While no frequency tagging studies to date have studied prosodic processing per se, it has been shown that imagined meter at 0.8 and 1.2 Hz is capable of eliciting significant EEG activity peaks in the absence of overt acoustic changes as well^68^. It is, therefore, highly plausible that a ½ sentence rate EEG peak in the 1 + 3 Syntax condition was driven by covert prosodic grouping.

Our Overt and Instructed Prosody conditions provided additional support for the role of prosody in the elicitation of low-frequency neurophysiological activity peaks. The OvP condition again elicited significant peaks at ½ sentence and sentence frequencies for both syntactic structures. While this result confirms that a prosodic contour is indeed sufficient to elicit the ½ sentence peak in absence of isochronous syntactic phrases (in 1 + 3 sentences), a concern is that this peak may be driven in a bottom-up fashion by acoustic cues already present in the speech signal (see Figs. 4a and 5a). However, as expected, interactions between syntactic structure (2 + 2 vs 1 + 3) and prosodic contour (ImplP vs W24) revealed that EEG activity peaks were differentially affected by the two prosodic contours. When the W24 contour aligned with syntax (in 2 + 2 sentences), the EEG signals at the ½ sentence frequency were significantly enhanced. When the contour was less aligned with syntactic phrasing (in 1 + 3) and arguably competed with the spontaneous prosodic pattern (eliciting the peak in the 1 + 3 ImplP condition), EEG peaks significantly decreased in amplitude (though as reported above, the effect at the ½ sentence frequency was smaller and only reached statistical significance, with *p* < 0.05, in the InstrP condition).

Taken together, these findings rule out any simple ‘additive’ contribution of bottom-up overt prosody tracking across both sentence types (2 + 2 and 1 + 3 Syntax). As predicted, 2 + 2 Syntax sentences received a boost for the ½ sentence frequency, because (i) the overt W24 prosodic contour partly added bottom-up prosodic entrainment activity in this frequency band (similar to the effects in our EEG pilot study), but also because (ii) the top-down syntactic phrasing of the 2 + 2 structure was supported by this prosodic contour. If we adopt the notion that posterior ½ sentence peaks reflect syntactic and anterior ½ sentence peaks reflect prosodic phrasing, the observed central distribution of this amplitude increase is in line with a combination of both effects in 2 + 2 Syntax sentences. By contrast, in 1 + 3 Syntax sentences, we observed reductions in peak amplitude, which can be explained by a conflict between two prosodic contours, namely the participants’ spontaneous phrasing pattern (observed in the ImplP condition) and the overt W24 contour. As described in the Methods section, a natural prosodic boundary marking—which we would expect to occur as a spontaneous implicit pattern in ImplP—typically involves duration changes such as syllable lengthening and pause insertion^20,47^. By contrast, our imposed W24 contour could not manipulate word or pause duration in order to be compatible with the requirements of frequency tagging (isochronous word duration). For these reasons, spontaneous prosody and W24 contours were incompatible, even though each of them promoted a 2 + 2 prosodic phrasing. Note that our hypothesized distinction in scalp topography between prosodic (frontal) and syntactic (more posterior) processing would predict that a prosodic conflict should lead to reduction of frontal activation, which is exactly what we observed. The sentence frequency peak for 1 + 3 Syntax sentences was more posterior (less frontal) in the OvP than in the ImplP condition (see voltage map in Fig. 6c).

This conflict found for OvP in 1 + 3 Syntax sentences prevented a boost at the ½ sentence frequency similar to that seen in 2 + 2 Syntax sentences. An important consequence was that the ½ sentence advantage for 1 + 3 Syntax sentences in the ImplP condition (i.e., a larger peak for 1 + 3 than 2 + 2 Syntax sentences) was not only lost, but reversed: with overt W24 prosody, 2 + 2 Syntax sentences had a significantly larger peak than 1 + 3 sentences. To summarize, the 2 + 2 Syntax condition profited from an overt W24 prosodic contour (enhanced peak amplitudes), whereas 1 + 3 sentences seemed to be more difficult to process (reduced amplitudes).

Data from our Instructed Prosody conditions are further in line with this view. Recall that in these trials, participants listened to the same sentences with neutralized prosodic cues that were used in the Implicit Prosody condition. Similar to a covert prosody paradigm previously studied using event-related potentials (ERP)^28^, participants had to silently imagine and superimpose the W24 prosodic contour they heard in the preceding overt prosody trial. While behavioural data demonstrated that participants still identified semantic outliers, the EEG data showed the effects of imagined, covert prosody. First of all, we again found the same elicited EEG peaks as in the other conditions, including the ½ sentence peak for 1 + 3 Syntax structures. As no prosodic cues were present in the speech signal, this finding not only illustrates that participants were successful in silently imagining and imposing the W24 contour (similar to the effects reported in non-linguistic research with instructed rhythms^68^) but also provides compelling evidence that the ½ sentence peak in 1 + 3 sentences can be elicited by a prosodic top-down mechanism, i.e., covert prosody. Intriguingly, the InstrP conditions replicated the differential effects of the W24 contour that we previously observed for overt prosody. When aligned with the syntactic structure (in 2 + 2 sentences), the EEG activity peaks were enhanced, whereas they were decreased in the case of prosodic misalignment with syntactic phrasing in 1 + 3 structures. The effects of overt and covert prosody were tightly linked, as reflected in their significant correlation in EEG power. Similar parallels between overt and covert prosodic processing have been found in numerous psycho- and neurolinguistic studies^20,24,26,29,80^.

The present study demonstrates the contribution of overt and covert prosody to low frequency cortical activity tracking sentence structure. Converging evidence across all three experimental conditions showed that (1) the 1 + 3 Syntax condition consistently elicited a ½ sentence peak in absence of a syntactic boundary, and that (2) adding a W24 prosodic contour both overtly through acoustic manipulation of the speech signal and covertly through instruction differentially changed this peak in 2 + 2 Syntax structures (peak enhancement) and 1 + 3 structures (peak reduction), respectively. However, while a contribution of bottom-up and top-down prosodic processing to the frequency peaks is convincing, we also found evidence for a role of top-down syntactic processes^14,15^. If prosody were all that matters, the differential influence of our prosodic manipulations on 2 + 2 versus 1 + 3 sentences would remain mysterious. In fact, neither a purely syntactic nor a purely prosodic account for frequency peaks would be compatible with our findings. Therefore, while our data relativize Ding et al.’^14^ conclusion of a purely syntactic effect—by demonstrating a clear role for prosody—our results in part support Ding and colleagues' claims by ruling out that prosody could account for all effects. Systematic associations between frontally distributed activation with prosodic processing and between more posteriorly distributed activation and syntactic processing suggests that these two sources can be distinguished from one another. Regardless of the exact mechanisms that underlie the neural activity tracking syntax and prosody in sentences, it is evident that the two mechanisms are interactive, and their integration is reflected in slow cortical responses.

The current study taps into the role of prosody in the elicitation of neural responses at sentence constituent frequencies. While we demonstrate the role of prosody in shaping these responses, future investigation will be needed to appreciate the role of other information types that are present in sentences and often correlated with syntactic structures (thematic roles, lexical semantics etc.). Importantly, while the frequency tagging technique has limited ecological validity, the original findings by Ding et al.^14,30^ were meant to have significant implications for the study of sentence processing beyond this specific experimental paradigm as well as for clinical practice. Our research questions these implications and calls for further investigations of online hierarchical structure processing and its integration with other layers of information in a sentence.

## Supplementary Information

Supplementary Information.

## Data Availability

The data that support the main findings of this study as well as the R scripts and the stimuli materials are available online: https://osf.io/qzbne/.
