# Supplementary material for: Overt and implicit prosody contribute to neurophysiological responses previously attributed to grammatical processing
Source: Sci Rep. 2022 Aug 30;12:14759. doi: 10.1038/s41598-022-18162-3 (PMC9427746; doi:10.1038/s41598-022-18162-3)
Supplement: Supplementary file 1 — Supplementary Information. [file 41598_2022_18162_MOESM1_ESM.docx]

**Overt and implicit prosody contribute to neurophysiological responses previously attributed to grammatical processing**

**- Supplementary Materials -**

Anastasia Glushko^1*^, David Poeppel^2,3,4^, and Karsten Steinhauer^1,5^

Affiliations:

^1^Centre for Research on Brain, Language and Music (Montreal, Canada)

^2^Department of Psychology, New York University (New York City, NY, USA)

^3^Ernst Struengmann Institute for Neuroscience (Frankfurt, Germany)

^4^Center for Language, Music, and Emotion (CLaME), New York

^5^School of Communication Sciences and Disorders, McGill University (Montreal, Canada)

* Contact information:

[anastasia.glushko@mail.mcgill.ca](mailto:anastasia.glushko@mail.mcgill.ca)

School of Communication Sciences and Disorders

2001 McGill College Avenue, Unit 800

H3A 1G1 Montreal, Quebec

Canada

## Manuscript 1: Supplementary materials

### Supplementary Materials A

**Full list of 1+3 Syntax sentences (English translations in parenthesis)**

Max baut das Haus. (Max is building the house.)

Hein fährt nach Bonn. (Hein is going to Bonn.)

Karl fand den Grund. (Karl found the reason.)

Fritz fliegt nach Neuss. (Fritz is flying to Neuss.)

Franz fragt nach Tee. (Franz is asking for tea.)

Ben fuhr mit mir. (Ben went with me.)

Till geht nach Rom. (Till is going to Rome.)

Kurt geht zum Turm. (Kurt is walking to the Tower.)

Jan giert nach Lob. (Yann is craving for praise.)

Lex greift nach Karl. (Lex is reaching out for Karl.)

Chris kennt das Buch. (Chris knows the book.)

Dirk kommt nach Laos. (Dirk comes to Laos).

Bert läuft zum Tor. (Bert is running to the gate.)

Tom lernt den Vers. (Tom is learning the verse.)

Lars mag das Bild. (Lars likes the picture.)

Phil maß das Bad. (Phil was measuring the bathroom.)

Hans mied den Rum. (Hans was avoiding the rum.)

Tim muss nach Kiel. (Timm has to go to Kiel.)

Klaus plant das Fest. (Klaus is planning the party.)

Knut putzt das Klo. (Knut is cleaning the toilet.)

Nick reist nach Lund. (Nick is travelling to Lund.)

Alf rennt zum Fluss. (Alf is running to the river.)

Paul riecht nach Rauch. (Paul is smelling like smoke.)

Mark ringt nach Luft. (Mark is gasping for air.)

Gert roch den Gin. (Gert smelled the gin.)

Ron ruft nach Lars. (Ron is calling Lars.)

Rolf rührt den Teig. (Rolf is stirring the dough.)

Jörg ruht seit neun. (Jorg has been resting since nine.)

Joern sah zum Baum. (Jorn looked at the tree.)

Ken schleicht zum Sitz. (Ken is sneaking to the seat.)

Kai spart das Geld. (Kai is saving the money.)

Rick spricht zum Volk. (Rick is speaking to the people.)

Sven stinkt nach Schweiss. (Sven stinks of sweat.)

Horst strebt nach Glück. (Horst is striving for happiness.)

Jens sucht das Mehl. (Jens is looking for flour.)

Nils wäscht das Hemd. (Nils is washing the shirt.)

Ralf wies zum Rad. (Rail is pointing to the bike.)

Kim wischt den Flur. (Kim is wiping the hallway.)

Wim zahlt das Bier. (Wim is paying for the beer.)

Phil zieht nach Prag. (Phil is moving to Prague.).

**Full list of 2+2 Syntax sentences**

Der Bär trinkt erst. (The bear is drinking first.)

Mein Baum steht da. (My tree is [standing] there.)

Das Bett riecht frisch. (The bed smells fresh.)

Das Blatt fliegt her. (The leaf is flying here.)

Mein Boot kippt um. (My boat tips over.)

Mein Boss spricht klar. (My boss speaks clearly.)

Dein Brot schmeckt schlecht. (Your bread tastes bad.)

Sein Buch schien cool. (His book seemed cool.)

Der Busch wächst schnell. (The bush is growing quickly.)

Ihr Chor sang schlimm. (Her choir sang poorly.)

Der Clown lacht lahm. (The clown is laughing lamely.)

Der Dieb lügt krass. (The thief is lying blatantly.)

Sein Fall lief schief. (His case went wrong.)

Sein Feld blüht schön. (His field is blossoming beautifully.)

Der Fisch schwimmt flink. (The fish is swimming nimbly.)

Mein Freund reist gern. (My friend likes to travel.)

Dein Holz brennt schnell. (Your wood burns fast.)

Sein Hund hört nicht. (His dog does not listen.)

Ihr Kerl spielt falsch. (Her guy is playing incorrectly.)

Ihr Kind schläft dort. (Her child is sleeping there.)

Ihr Kleid ist naß. (Her dress is wet.)

Ihr Koch backt nie. (Her cook never bakes.)

Mein Kopf juckt rechts. (My head is itching on the right.)

Sein Licht scheint schwach. (His light seems weak.)

Ihr Mann sieht kaum. (Her husband barely sees.)

Das Paar wohnt links. (The couple lives on the left.)

Sein Pferd trabt schwer. (His horse is trotting heavily.)

Mein Pool roch fies. (My pool smelled nasty.)

Das Reh fällt hin. (The is falling.)

Das Schaf war naß. (The sheep was wet.)

Dein Schal weht rum. (Your scarf is blowing around.)

Der Schnee schmilzt früh. (The snow metls early.)

Dein Schrank wog viel. (Your dresser weighed a lot.)

Dein Sohn ritt fort. (Your son rode away.)

Dein Song klingt nett. (Your song sounds nice.)

Das Team malt selbst. (The team paints itself.)

Mein Vieh ging ein. (My kettle is dying off.)

Der Wolf biss oft. (The wolf bit often.)

Das Zelt blieb hier. (The tent stayed here.)

Sein Ziel hing hoch. (His goal was [hung] high.)

**Outlier sentences: 1+3 Syntax**

Max baut den Rauch. (Max is building the smoke.)

Rolf rührt das Haus. (Rolf is mixing the house.)

Jan giert nach Kiel. (Yann is craving Kiel.)

Lex greift nach Prag. (Lex is attacking Prague.)

Dirk kommt nach Tee. (Dirk is coming to tea.)

Kim wischt das Glück. (Kim is washing happiness.)

Nils wäscht das Mehl. (Nils is washing the flour.)

Hein fährt nach Grund. (Hein is going to the reason.)

Knut putzt den Vers. (Knut is cleaning the verse.)

Kurt geht nach Lob. (Kurt is going to the praise.)

Gert roch das Fest. (Gert smelled the celebration.)

Tom lernt das Hemd. (Tom is learning the shirt.)

**Outlier sentences: 2+2 Syntax**

Dein Brot sang schlimm. (Your bread sang poorly.)

Der Bär blüht schön. (The bear is blossoming beautifully.)

Mein Boot trinkt erst. (My boat is drinking first.)

Dein Schal trabt schwer. (Your scarf is trotting heavily.)

Das Zelt lacht lahm. (The tent is laughing lamely.)

Der Busch schläft dort. (The bush is sleeping there.)

Dein Holz lügt krass. (Your wood is lying blatantly.)

Sein Fall biss oft. (His fall bit often).

Der Wolf schmilzt früh. (The wolf melts early.)

Sein Feld wohnt links. (His field lives on the left.)

Sein Licht juckt rechts. (His light is itching on the right.)

Das Bett spielt falsch. (The bed is playing incorrectly.)

As part of a different experiment not reported on here, we asked six native speakers of German to rate the sentences used in the present study (but with a somewhat different prosodic contour applied to them) as semantically plausible or semantically plausible. The sentences were listened to one-by-one, with the plausibility question after each sentence. The results indicated that only one outlier sentence (“Gert roch das Fest”) was primarily rated as being plausible; the vast majority of the outlier sentences were unanimously rated as semantically implausible.

**Stimuli intelligibility across OvP conditions and for the ImplP sentences**.

Participants were presented with all 104 sentences one-by-one. They could listen to every sentence maximum twice and had to type in what they understood. Response was considered correct if the sentence typed in by the participant was identical to the one presented, or if there were minor orthographical errors and/or if one proper name was mistaken by another one while not changing the meaning of the sentence. The EEG (and behavioural) data from the participant who scored 49 out of 104 on the ImplP sentences were not analyzed. The results are presented in Supplementary Figure S1. Note that the slight difference in ranking between ImplP and OvP sentences could be caused by the fact that participants in the OvP intelligibility pilot study were recruited separately from the main study and did not get to read the full list of sentences prior to the sentence intelligibility test. Thirty-seven participants performed the ImplP sentence intelligibility task, however, only 25 of them went through the full study.

**Supplementary Figure S1. Stimuli intelligibility scores across experimental conditions: (a) Implicit Prosody and (b) Overt Prosody sentences.**

###
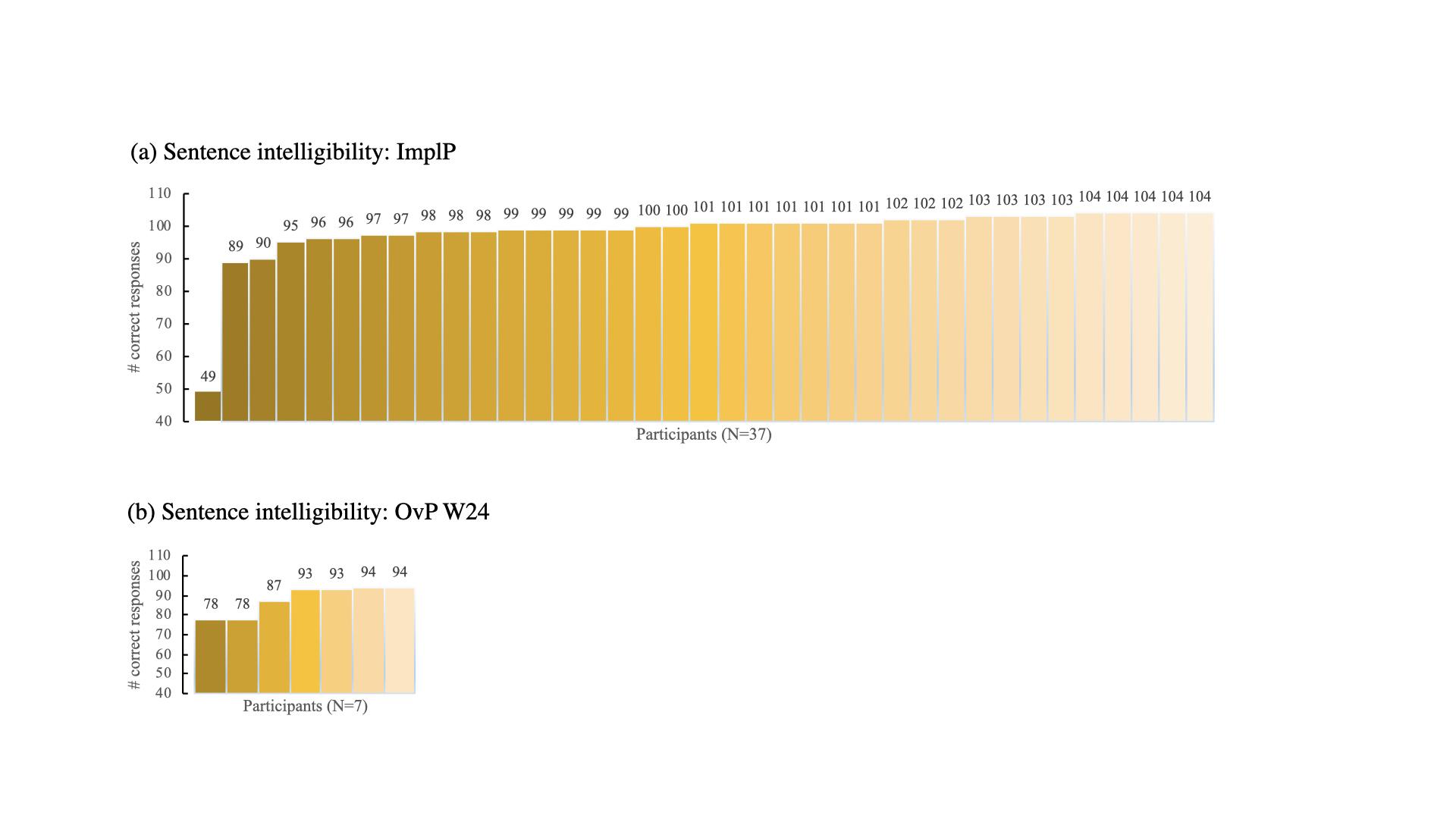


**Supplementary Figure S2. Proportions of correct responses in the plausibility rating task across conditions (as requested by one of the reviewers; please see the results of modeling these results with linear mixed-models in the main body of the manuscript).** Bold horizontal lines within the box plots represent medians across subjects, the box covers the interquartile range (IQR), while the whiskers extend to the values equal to the closest edge of the IQR + 1.5 × IQR; the dots reflect the remaining outliers.

**
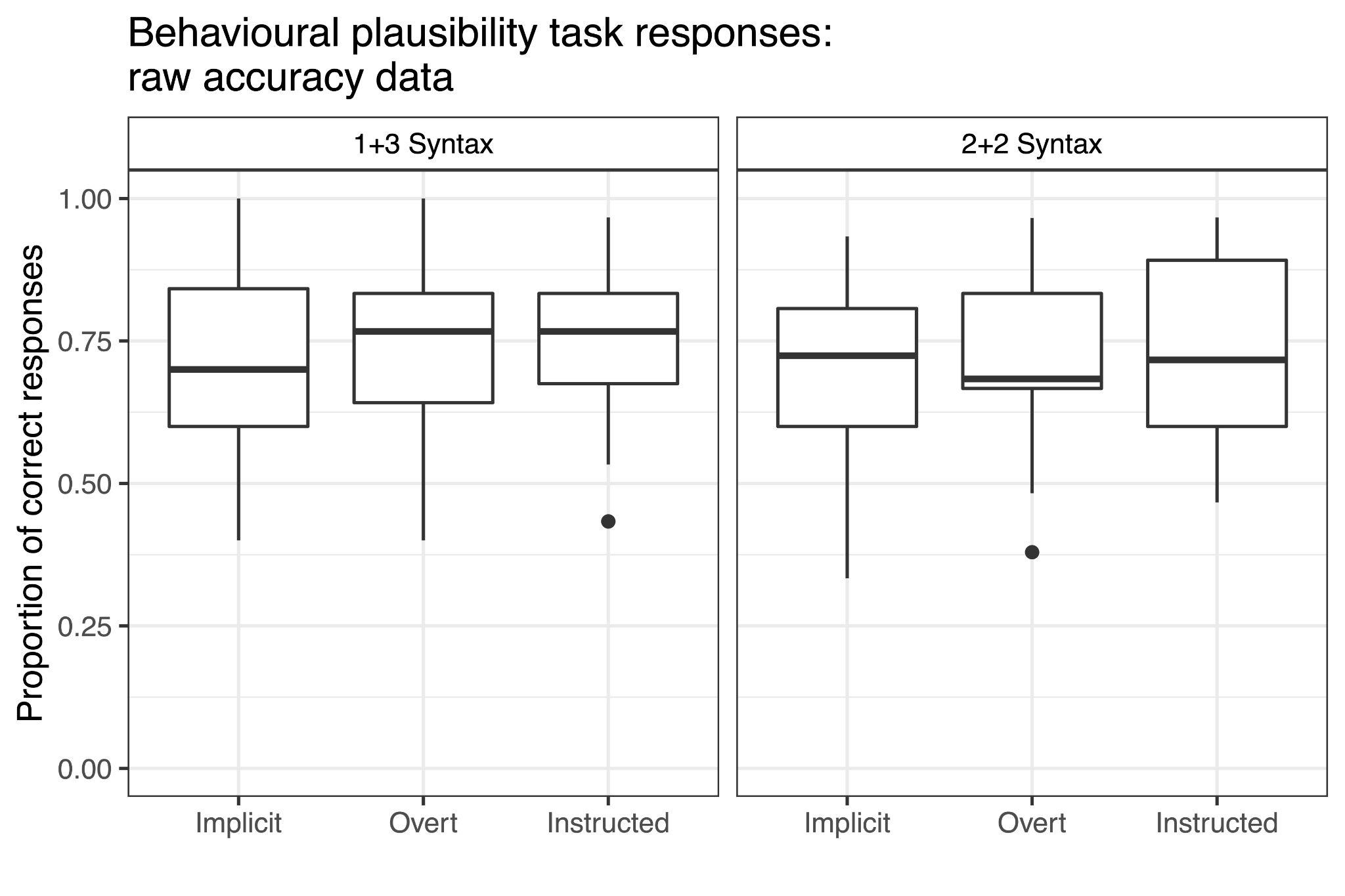
**

### Supplementary Materials B

**Control experiment: Non-syntactic grouping**

Ding and colleagues (2016) investigated the processing of 1+3 Syntax structures (for example, fry_1_ | to-ma-to_3_; see main text for details) using frequency tagging with MEG. To ensure that the results from this condition will be comparable to their data when EEG is used, we conducted a control experiment using non-syntactic rhythmic grouping. The reason for using non-syntactic grouping is that with natural language materials, many layers of information (e.g., semantics, syntax, prosody) can contribute to chunking of words into larger constituents. In our case, only one type of manipulation (rhythmic grouping differences) was ensured by using nonword materials. We compared processing of 2+2 and 1+3 grouping created by manipulating the average fundamental frequency at which the nonword was presented, resulting in artificially “sung” stimuli. Sequences made of four monosyllabic nonwords each contained two chunks: one presented with the average pitch of 123.47 Hz, corresponding to the B of the second octave, and the other one with the pitch of 164.81 Hz, corresponding to the E of the third octave (see below).

**Methods**

Participants. Eleven German native speakers participated in the experiment (age range: 20-35 years, mean age = 26; 6 women, 5 men). Participant inclusion criteria were identical to the ones in the main study.

Materials. We recorded a German native speaker pronouncing fifteen monosyllabic non-words that were phonotactically correct in German. The pitch in them was flattened, and the intensity was normalized in Praat (Boersma & Weenink, 2019) to 70 dB. The length of each nonword was manually adjusted to 250 ms. The nonwords were concatenated into sequences of four in a pseudorandom manner: within each sequence, no vowel or consonant was repeated, and no real words were created through concatenation. The 4-nonword sequences were concatenated into trials comprising 12 sequences each (48 nonwords). Each sequence lasted for one second, making trials 12 seconds long. No pauses were introduced at any time point within trials. Given the length of each nonword in this experiment was 250 ms, the nonword frequency corresponded to 4 Hz, the ½ sequence frequency to 2 Hz, and the sequence frequency to 1 Hz.

For each 4-nonword sequence, pitch was manipulated in a way specific to either the 1+3 or the 2+2 grouping. In the case of the 1+3 grouping, the first nonword was assigned a pitch of 123.47 Hz, and nonwords 2, 3, and 4 were assigned a pitch of 164.81 Hz. In the 2+2 grouping, the pitch of 123.47 Hz was imposed in Praat on the first two nonwords, and the pitch of 164.82 on nonwords 3 and 4. Note that sound intensity was controlled for, and sound intensity fluctuations were only observed at the nonword (i.e., the syllabic) rate (see left panel of Supplementary Figure S3).

To keep participants’ attention, similar to the main study, we used an outlier detection task. Thirty trials per grouping were presented to each participant. In twenty two of these trials, all nonword sequences had the same (either 1+3 or 2+2) grouping. The 8 trials contained rhythmic outliers: either a 1+3 grouping sequence was inserted in a trial of 2+2 grouping sequences, and vice versa.

Procedure. This experiment was part of a series of pilot tests administered with the same group of participants. In total, participants came to the lab for approximately 4 hours. After filling out behavioural questionnaires (identical to the ones in the main study), participants were brought into the EEG booth, with the EEG session lasting for approximately 2 hours with several major breaks between tests. Participants were told that the sequences of syllables would form patterns. After each trial they were asked to respond if the main pattern within a trial has ever been broken or not. Trials were presented in blocks of 30, with blocks containing correct and outlier trials for one type of grouping (either 1+3 or 2+2).

EEG recording and processing. See main study for details. In contrast to the main study, single bin values in this experiment were calculated at the frequency of 0.0833 Hz (different from the main study due to the sequences of nonwords being shorter in this experiment).

Statistical analysis. We computed the average accuracy and the d-prime for the behavioural data in this pilot experiment in R (R Core Team, 2018). The EEG power was analyzed separately for the 1+3 and the 2+2 grouping using bias-corrected and accelerated bootstrap tests contrasting the response at either the ½ sequence or the sequence frequency with the noise (average EEG power at neighbouring bins comprising 0.5 Hz prior as well as 0.5 Hz following the target frequency). The p-values were corrected for multiple comparisons using the Bonferroni method.

**Results and discussion**

The accuracy on the outlier detection task was 66.1%, with the mean d-prime of 1.175 meaning participants did distinguish between the two types of rhythmic grouping. The EEG findings (Supplementary Figure S3) were in line with the behavioural data. The EEG responses at the sequence rate (1 Hz) were significantly different from noise in both 1+3 and 2+2 grouping types (both p-values < .001). At the ½ sequence rate, as predicted based on the MEG results in Ding and colleagues’ study (2016), the EEG power was only significant from noise in the 2+2 grouping (p < .001). These results suggest that 1+3 grouping can be used in EEG frequency tagging studies as a control condition for constructions with 2+2 grouping.


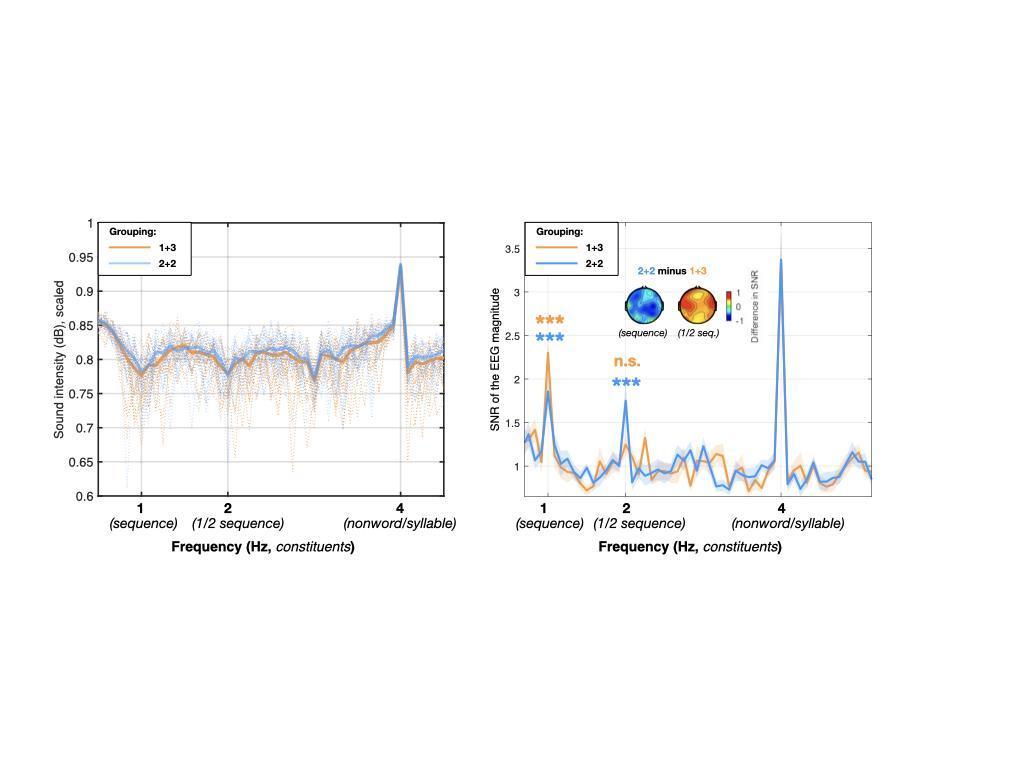


**Supplementary Figure S3. Sound intensity envelope and the EEG magnitude spectra for 1+3 and 2+2 nonword grouping based on pitch changes (“sung” groupings).** On the left is the sound intensity envelope calculated per trial, converted to a logarithmic decibel scale, and scaled from 0 to 1. The envelope was computed for each trial separately (thin dotted lines) and then averaged across trials (thick solid lines). On the right is the EEG magnitude spectrum from 11 participants in this experiment. Note that while the EEG responses at the frequency of a full sequence are significantly greater than noise in both conditions, while at the ½ sequence frequency this is only the case for the 2+2 grouping. The scalp maps depict distribution of the differences between EEG magnitude SNR in the two conditions (2+2 minus 1+3 grouping) across electrodes, separately for the sequence and the ½ sequence frequency. ^***^p<0.001.

**References**

Ding, N., Melloni, L., Zhang, H., Tian, X., & Poeppel, D. (2016). Cortical tracking of hierarchical linguistic structures in connected speech. Nature neuroscience, 19(1), 158.

R Core Team (2018). R: A language and environment for statistical computing. R Foundation for Statistical Computing, Vienna, Austria. URL [https://www.R-project.org/](https://www.r-project.org/).

### Supplementary Materials C

**No Semantic Task condition**

Similarly to the Overt and Instructed Prosody conditions in the main part of the study, participants listened to OvP and InstrP trials, however, in the No Semantic Task condition, outlier trials were excluded. Participants’ task was to listen to the OvP trial passively, and then listen to the InstrP trial while imagining the intonation present in the preceding OvP sentences.

**Methods**

We analyzed a subset of 10 participants who were presented with the prosodic contour investigated in the frame of the current study and had sufficient artifact-free EEG data (age range: 20-37 years, mean age = 26, age SD = 6; 5 females, 5 males). The data were analyzed for the 1+3 Syntax W24 (N=10) condition only (the one for which the data of at least 10 participants were available). The EEG effects in the No Semantic Task were contrasted with the corresponding conditions in the main part of the study. A generalized linear model (Gamma family with identity link) was fitted for normalized EEG power analysis. The factors tried for the inclusion in the model being: Task (Dual task vs. No Semantic Task), Prosody (Overt vs. Covert) × Frequency; Laterality, Anteriority, and all possible interactions between them. The rest of the methodological aspects of the No Semantic Task part of the experiment were identical to those in the OvP and InstrP conditions discussed in the main body of the manuscript.

**Results and discussion**

We used the No Semantic Task condition to investigate the effect of task-driven processing on the results of the OvP and InstrP conditions in the main part of the study. We presumed that the EEG responses in the No Semantic Task condition would reflect the shift of attention from semantic information to prosody, and this would be especially prominent in the InstrP condition where participants had to actively perform a prosodic task. We analyzed the data from the 1+3 Syntax sentences and found that the signal at ½ sentence and sentence frequencies was significantly larger than noise in the overt and covert W24 condition (all p-values < .001, except NoSem InstrP, sentence frequency: p = .006 and NoSem OvP, sentence frequency: p = .002; see Supplementary Figure S4).


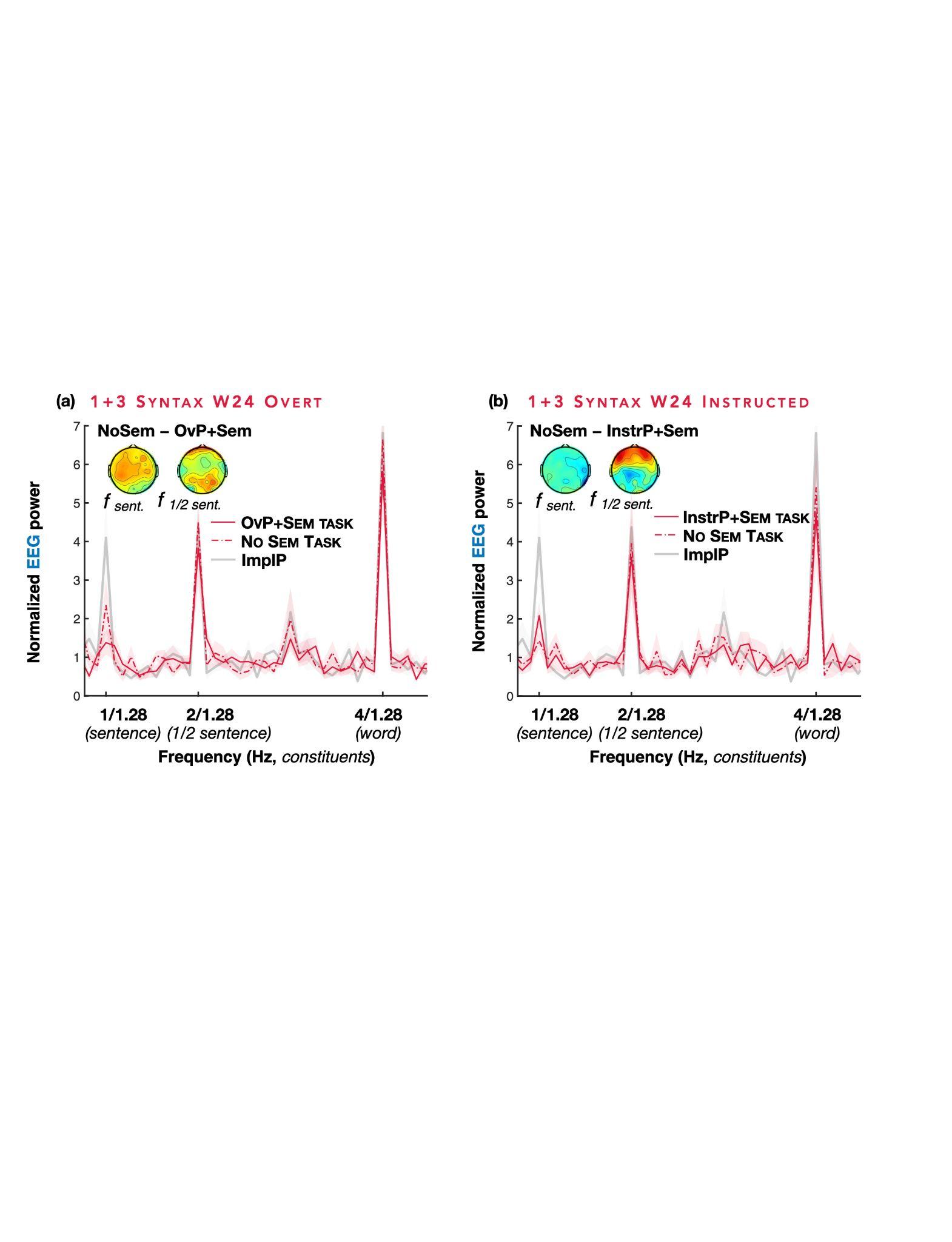


**Supplementary Figure S4. EEG power in the No Semantic Task condition: 1+3 Syntax sentences with overt (a) and instructed (b) W24 prosodic contour.** Data from 10 participants per condition are plotted against the data from the same participants in the No Semantic Task (dashed-dotted red lines), the OvP and InstrP conditions with a semantic task (solid red lines), and the Implicit Prosody conditions (bold grey lines). The Implicit Prosody condition is added to the graph to demonstrate the absence of the qualitative differences between the data of the subset of participants and the data in the main Implicit Prosody condition (however, the Implicit Prosody condition was not included in the statistical analysis, for which only the comparison between the OvP and InstrP with and without the a semantic task was quantified). The lines in the spectrum plots reflect group averages of the EEG magnitude SNR in the frequency domain, with the shaded area depicting standard errors of the mean. Scalp maps represent the scalp distribution of difference between the effects in the No Semantic Task conditions and OvP and InstrP with a semantic task.

We expected that effects related to prosody would be enhanced in the No Semantic Task conditions compared to the conditions with a semantic task, and the effects related to semantic sentence integration reduced. The only effect supporting this idea was the somewhat enhanced EEG power in the absence of any semantic task enhanced EEG responses in the OvP condition (Task × Prosody: β = -0.223, SE = 0.076, p = .003; OvP without semantic task – OvP with semantic task: β = 0.561, SE = 0.233, p = .066). This effect was, contrary to our expectations, not seen in the InstrP condition, pointing to participants focussing on imagining the prosodic contour equally in the conditions with and without semantic tasks. Given the low number of participants in this part of the study, future research should continue testing task effects in frequency tagging paradigms.
